# Enhancer hijacking determines extrachromosomal circular *MYCN* amplicon architecture in neuroblastoma

**DOI:** 10.1038/s41467-020-19452-y

**Published:** 2020-11-16

**Authors:** Konstantin Helmsauer, Maria E. Valieva, Salaheddine Ali, Rocío Chamorro González, Robert Schöpflin, Claudia Röefzaad, Yi Bei, Heathcliff Dorado Garcia, Elias Rodriguez-Fos, Montserrat Puiggròs, Katharina Kasack, Kerstin Haase, Csilla Keskeny, Celine Y. Chen, Luis P. Kuschel, Philipp Euskirchen, Verena Heinrich, Michael I. Robson, Carolina Rosswog, Joern Toedling, Annabell Szymansky, Falk Hertwig, Matthias Fischer, David Torrents, Angelika Eggert, Johannes H. Schulte, Stefan Mundlos, Anton G. Henssen, Richard P. Koche

**Affiliations:** 1 Department of Pediatric Oncology/Hematology, Charité—Universitätsmedizin Berlin, Augustenburger Platz 1, 13353 Berlin, Germany; 2 RG Development & Disease, Max Planck Institute for Molecular Genetics, Ihnestraße 63-73, 14195 Berlin, Germany; 3 Institute for Medical and Human Genetics, Charité—Universitätsmedizin Berlin, Augustenburger Platz 1, 13353 Berlin, Germany; 4 Berlin-Brandenburg Center for Regenerative Therapies (BCRT), Charité—Universitätsmedizin Berlin, Augustenburger Platz 1, 13353 Berlin, Germany; 5 Experimental and Clinical Research Center (ECRC), Max Delbrück Center for Molecular Medicine and Charité—Universitätsmedizin Berlin, Lindenberger Weg 80, 13125 Berlin, Germany; 6 Barcelona Supercomputing Center, Joint BSC-CRG-IRB Research Program in Computational Biology, Jordi Girona 29, 08034 Barcelona, Spain; 7 German Cancer Consortium (DKTK), partner site Berlin, and German Cancer Research Center DKFZ, Im Neuenheimer Feld 280, 69120 Heidelberg, Germany; 8 Department of Neurology with Experimental Neurology, Charité—Universitätsmedizin Berlin, Charitéplatz 1, 10117 Berlin, Germany; 9 Berlin Institute of Health, Anna-Louisa-Karsch-Str. 2, 10178 Berlin, Germany; 10 Department of Computational Molecular Biology, Max Planck Institute for Molecular Genetics, Ihnestraße 63-73, 14195 Berlin, Germany; 11 Department of Experimental Pediatric Oncology, University Children’s Hospital of Cologne and Center for Molecular Medicine Cologne (CMMC), University of Cologne, Kerpener Straße 62, 50937 Köln, Germany; 12 Institució Catalana de Recerca i Estudis Avançats (ICREA), Passeig Lluís Companys 23, 08010 Barcelona, Spain; 13 Center for Epigenetics Research, Memorial Sloan Kettering Cancer Center, 430 East 67th Street, New York, NY 10065 USA

**Keywords:** Cancer genomics, Paediatric cancer, Epigenomics

## Abstract

*MYCN* amplification drives one in six cases of neuroblastoma. The supernumerary gene copies are commonly found on highly rearranged, extrachromosomal circular DNA (ecDNA). The exact amplicon structure has not been described thus far and the functional relevance of its rearrangements is unknown. Here, we analyze the *MYCN* amplicon structure using short-read and Nanopore sequencing and its chromatin landscape using ChIP-seq, ATAC-seq and Hi-C. This reveals two distinct classes of amplicons which explain the regulatory requirements for *MYCN* overexpression. The first class always co-amplifies a proximal enhancer driven by the noradrenergic core regulatory circuit (CRC). The second class of *MYCN* amplicons is characterized by high structural complexity, lacks key local enhancers, and instead contains distal chromosomal fragments harboring CRC-driven enhancers. Thus, ectopic enhancer hijacking can compensate for the loss of local gene regulatory elements and explains a large component of the structural diversity observed in *MYCN* amplification.

## Abstract (web summary)

MYCN amplification is common in neuroblastomas. Here the authors analyse the MYCN amplicon structure and its epigenetic regulation by integrating short- and longread genomic and epigenomic data and find two classes of MYCN amplicons in neuroblastomas, one driven by local enhancers and the other by hijacking of distal regulatory elements.

## Introduction

Oncogene amplification is a hallmark of cancer genomes. It leads to excessive proto-oncogene overexpression and is a key driver of oncogenesis. The supernumerary gene copies come in two forms: (i) self-repeating arrays on a chromosome (homogeneously staining regions, HSR) and (ii) many individual circular DNA molecules (extrachromosomal DNA, ecDNA, alias double minute chromosomes, dmin)^1^. EcDNA can arise during genome reshuffling events like chromothripsis and are subsequently amplified^2,3^. This partially explains why ecDNA can consist of several coding and non-coding distal parts of one or more chromosomes^4^. Over time, amplified DNA acquires additional internal rearrangements as well as coding mutations, which can confer adaptive advantages such as resistance to targeted therapy^5–7^. EcDNA reintegration into chromosomes can lead to intrachromosomal amplification as HSRs^8,9^ and act as a general driver of genome remodeling^10^. Our knowledge of the functional relevance of non-coding regions co-amplified on ecDNA, however, is currently limited.

*MYCN* amplification is a prototypical example of a cancer-driving amplification. The developmental transcription factor was identified as the most commonly amplified gene in a recent pediatric pan-cancer study^11^. Its most prominent role is in neuroblastoma, a pediatric malignancy of the sympathetic nervous system. *MYCN* amplification characterizes one in six cases and confers dismal prognosis^12^. In contrast to long-term survival of more than 80% for non-amplified cases, 5-year overall survival is as low as 32% for *MYCN*-amplified neuroblastoma^12^. In these cases, *MYCN* amplification is likely an early driver of neuroblastoma formation. Indeed, *MYCN* overexpression is sufficient to induce neuroblastic tumor formation in mice^13,14^. Despite its central role in neuroblastoma biology, the epigenetic regulation of *MYCN* is incompletely understood.

Recently, studies have identified a core regulatory circuit (CRC) including half a dozen transcription factors that drive a subset of neuroblastomas with noradrenergic cell identity, including most *MYCN*-amplified cases^15–18^. The epigenetic landscape around *MYCN* is less well characterized. In part, this is due to the structural complexity of *MYCN* amplicons and difficulties in interpreting epigenomic data in the presence of copy-number variation. Recent evidence has emerged suggesting that local enhancers may be required for proto-oncogene expression on amplicons^19^. Structural rearrangements can also juxtapose ectopic enhancers to proto-oncogenes and thereby drive aberrant expression, a phenomenon known as enhancer hijacking in several pediatric tumors^20–24^. Here, we seek out to identify key regulatory elements near *MYCN* in neuroblastoma by integrating short- and long-read genomic and epigenomic data from neuroblastoma cell lines and primary tumors. We investigate the activity of regulatory elements in the context of *MYCN* amplification and characterize the relationship between amplicon structure and epigenetic regulation. This reveals the retention of local CRC-driven enhancers on the *MYCN* amplicon in the majority of cases. When such local elements are not co-amplified, however, amplicons are structurally complex and distal elements are combined to form novel gene-regulatory neighborhoods.

## Results

### Defining the local enhancer landscape of *MYCN*

**Fig. 1:**
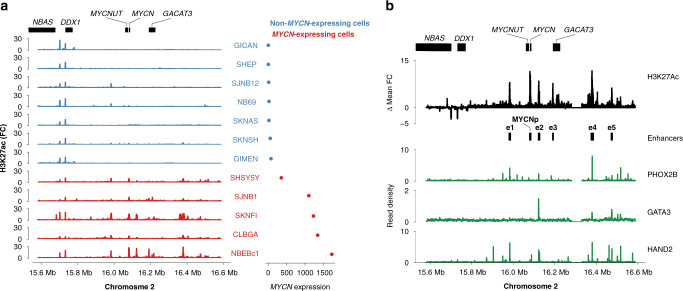
Five enhancers are specifically found in *MYCN*-expressing neuroblastoma cells. **a** H3K27ac ChIP-seq fold change over input (left) and size-factor normalized *MYCN* expression as determined from RNA-seq for 12 non-MYCN-amplified neuroblastoma cell lines. **b** Aggregated H3K27ac signal of *MYCN*-expressing compared to non-expressing cells (top, black; MYCNp, *MYCN* promoter; e1–e5, *MYCN*-specific enhancers). PHOX2B, GATA3, and HAND2 core regulatory circuit transcription factor ChIP-seq in an *MYCN*-expressing neuroblastoma cell line (green, CLB-GA). Source data are provided as a Source Data file.

Acetylation at the 27th lysine residue of the histone H3 protein (H3K27ac) characterizes active chromatin at promoters and enhancers^25^. In order to identify candidate active regulatory elements near *MYCN*, we examined public H3K27ac chromatin immunoprecipitation and sequencing (ChIP-seq) and RNA sequencing (RNA-seq) data from 25 neuroblastoma cell lines^15^. ChIP-seq data for amplified genomic regions are characterized by a very low signal-to-noise ratio, which has complicated their interpretation in the past^16^. We therefore focused our analysis on 12 cell lines lacking *MYCN* amplifications but expressing *MYCN* at different levels, allowing for the identification of *MYCN*-driving enhancers in neuroblastoma. Comparison of composite H3K27ac signals of *MYCN*-expressing vs. non-expressing cell lines identified at least five putative enhancer elements (e1–e5) that were exclusively present in the vicinity of *MYCN* in cells expressing *MYCN*, thus likely contributing to *MYCN* regulation (Fig. 1 and Supplementary Fig. 1a). Consistent with differential RNA expression, a strong differential H3K27ac peak was identified spanning the *MYCN* promoter and gene body (MYCNp; Fig. 1). The identified enhancers were not active in developmental precursor cells such as embryonic stem cells, neuroectodermal cells, neural crest cells, or fetal adrenal cells (Supplementary Fig. 1b), suggesting these enhancers were specific for later stages of sympathetic nervous system development or neuroblastoma. Transcription factor ChIP-seq in *MYCN*-expressing cells confirmed that four of the enhancers (e1, e2, e4, and e5) were bound by each of three noradrenergic neuroblastoma core regulatory circuit transcription factors (PHOX2B, HAND2, GATA3; Fig. 1b). All but enhancer e3 harbored binding motifs for the remaining members of the CRC (ISL1, TBX2, ASCL1; Supplementary Fig. 1c) for which ChIP-seq data were unavailable. Additionally, all enhancers contained binding motifs for TEAD4, a transcription factor implicated in a positive feedback loop with MYCN in *MYCN*-amplified neuroblastoma^26^. Two of the enhancers (e1 and e2) also harbored canonical E-boxes, suggesting binding of MYCN at its own enhancers (Supplementary Fig. 1c). Taken together, a common set of CRC-driven enhancers is found uniquely in *MYCN*-expressing neuroblastoma cells, indicating that *MYCN* expression is regulated by the CRC.

### Enhancer selection explains MYCN amplicon boundaries

**Fig. 2:**
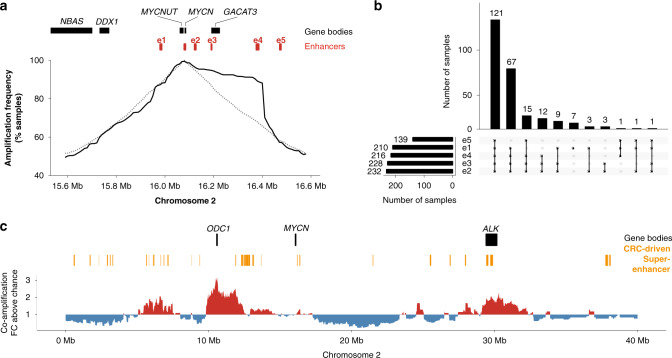
Local enhancer e4 is significantly co-amplified with *MYCN*. **a** Co-amplification frequency of the immediate *MYCN* neighborhood measured using copy-number profiles from 240 *MYCN*-amplified neuroblastomas (solid line) compared to the expected co-amplification frequencies for randomized *MYCN*-containing amplicons (dashed line). **b** Upset plot showing the co-amplification patterns of all five *MYCN*-specific local enhancers identified in neuroblastoma. **c** Enrichment for co-amplification with *MYCN* of genomic regions on 2p (red, co-amplification more frequent than expected by chance; blue, co-amplification less frequent than expected by chance). Source data are provided as a Source Data file.

*MYCN* is expressed at the highest levels in neuroblastomas harboring *MYCN* amplifications, with a strong effect of genomic copy number on expression levels (Supplementary Fig. 1d, e). It is unclear, however, to what extent enhancers are required for sustained *MYCN* expression on *MYCN*-containing amplicons. To address this, we mapped amplified genomic regions in a meta-dataset of copy-number variation in 240 *MYCN*-amplified neuroblastomas^27^. This revealed an asymmetric pattern of *MYCN* amplification (Fig. 2a and Supplementary Fig. 2). Intriguingly, a 290 kb region downstream of *MYCN* was co-amplified in more than 90% of neuroblastomas, suggesting that *MYCN* amplicon boundaries were not randomly distributed, which is in line with recent reports using a smaller tumor cohort^19^. Notably, the consensus amplicon boundaries did not overlap with common fragile sites (Supplementary Fig. 2g), challenging a previous association found in 24 neuroblastoma cell lines and tumors^28^. Regions of increased chromosomal instability alone are therefore unlikely to explain amplicon boundaries. Strikingly, several *MYCN*-specific enhancers were found to be commonly co-amplified (Fig. 2b). The distal *MYCN*-specific CRC-driven enhancer, e4, was part of the consensus amplicon region in 90% of cases. Randomizing amplicon boundaries around *MYCN* showed that e4 co-amplification was significantly enriched on *MYCN* amplicons (empirical *P* = 0.0003). Co-amplification frequency quickly dropped downstream of e4, suggesting that *MYCN*-specific, CRC-driven enhancers are a determinant of *MYCN* amplicon structure and may be required for *MYCN* expression, even in the context of high-level amplification.

Considering that *MYCN* is amplified in many pediatric cancer entities that differ in chromatin landscape, we hypothesized that *MYCN* amplicon structure should also differ between cancer entities. To test this, we inspected the amplicon architecture in a cohort of sonic hedgehog-driven medulloblastomas (SHH-MB) and Group 4 medulloblastomas (GROUP4-MB)^29^, which often harbor *MYCN* amplifications and are commonly thought to originate from different precursor cell types^30^. In line with our model of tissue-specific enhancer co-amplification, *MYCN* amplicon structure differed between medulloblastomas and neuroblastomas (Supplementary Fig. 3a). *MYCN* amplicon distributions also differed between SHH-MB and GROUP4-MB (Supplementary Fig. 3b). A SHH-MB-specific super-enhancer (SE) > 350 kb downstream of *MYCN* was co-amplified in 8/9 cases, indicating selection. GROUP4-MB lack *MYCN*-driving SEs and are characterized by several enhancers close to *MYCN*. At least one of these local enhancers was co-amplified in 11/12 cases. Thus, tissue-specific enhancers are a determinant of *MYCN* amplicon structure and may be required for *MYCN* expression in various tumor entities.

### Distal super-enhancer co-amplification with *MYCN*

We and others have previously described chimeric *MYCN* amplicons^10^ containing distal chromosomal fragments. We therefore systematically inspected *MYCN*-distal regions on chromosome 2 for signs of co-amplification. Distinct regions were statistically enriched for co-amplification with *MYCN* (Fig. 2c). In line with previous reports^31^, significant co-amplification of 19 protein-coding genes, including known neuroblastoma drivers such as *ODC1*, *GREB1,* and *ALK* occurred in *MYCN*-amplified neuroblastoma. Notably, co-amplification of distal CRC-driven SEs occurred in 23.3% of samples. Seven specific CRC-driven SEs were significantly co-amplified more often than expected by chance. Most of these SEs were found in gene-rich regions, making it difficult to discern whether genes or regulatory elements were driving co-amplification. One significantly co-amplified CRC-driven SE, however, was found in a gene-poor region in 2p25.2, where most co-amplified segments did not overlap protein-coding genes (Fig. 2c). This led us to ask whether hijacking of such distal regulatory elements could explain co-amplification with *MYCN*.

### Enhancers remain functional on *MYCN* amplicons

**Fig. 3:**
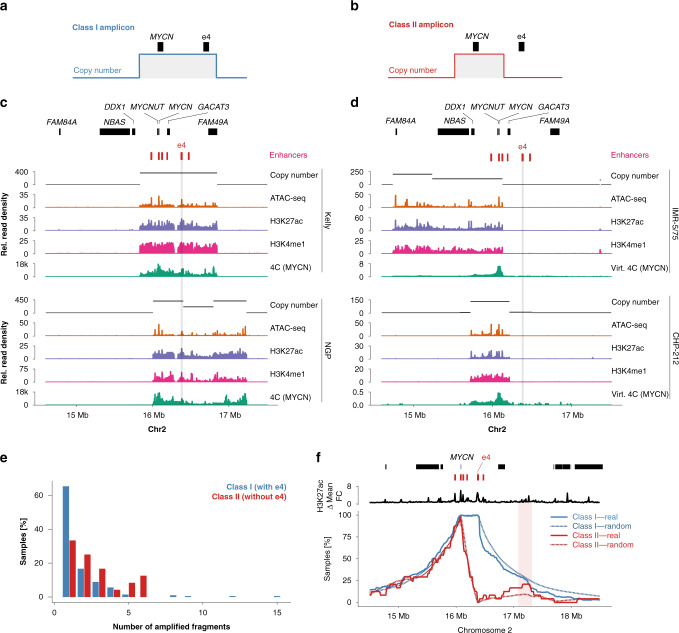
Two classes of MYCN amplicons can be identified in neuroblastoma. Schematic representation of class I (**a**) and class II (**b**) *MYCN* amplicons. **c** Copy-number profile (black), ATAC-seq (orange), H3K27ac ChIP-seq (purple), H3K4me1 ChIP-seq (pink), and 4C (*MYCN* promoter as the viewpoint; green) for two neuroblastoma cell lines with class I amplicons, co-amplifying the e4. **d** Copy-number profile (black), ATAC-seq (orange), H3K27ac ChIP-seq (purple), H3K4me1 ChIP-seq (pink), and virtual 4C (*MYCN* locus as the viewpoint; green) for two neuroblastoma cell lines class II amplicons, not co-amplifying e4. **e** Number of non-contiguous amplified fragments in class I samples (*N* = 216) vs. class II samples (*N* = 24). **f** Amplicon boundary frequency relative to gene and enhancer positions in class I vs. class II amplicons compared to random amplicon boundary frequencies. Source data are provided as a Source Data file.

Based on our amplicon boundary analysis, two classes of *MYCN* amplicons could be distinguished in neuroblastoma: (i) amplicons containing local *MYCN*-specific enhancers, including e4 (here referred to as class I amplicons; Fig. 3a) and (ii) amplicons lacking local *MYCN*-specific enhancers, and at least lacking e4 (referred to as class II amplicons; Fig. 3b). To determine whether co-amplified enhancers were active, we acquired genomic (long- and short-read whole-genome sequencing) and epigenomic (Assay for Transposase-Accessible Chromatin using sequencing, ATAC-seq, and mono-methylation at the fourth lysine residue of the histone H3, H3K4me1, and H3K27ac ChIP-seq) data for two neuroblastoma cell lines with class I amplicons (Kelly and NGP) and two neuroblastoma cell lines with class II amplicons (IMR-5/75 and CHP-212). Notably, H3K27ac signal-to-noise ratio was lower on *MYCN* amplicons than in non-amplified regions. While the fraction of reads in peaks was similar across amplicons and randomly drawn regions, we observed more peaks on the amplicon than for non-amplified regions (Supplementary Fig. 4). These peaks were characterized by a lower relative signal compared to the amplicon background signal, indicating a larger variety of active regulatory regions on different *MYCN* amplicons. Using nanopore long-read-based de novo assembly, we reconstructed the *MYCN* neighborhood, confirming that *MYCN* and e4 were not only co-amplified in class I amplicons, but also lacked large rearrangements, which could preclude enhancer–promoter interaction (Supplementary Figs. 5 and 6). Enhancer e4 was characterized by increased chromatin accessibility and active enhancer histone marks as determined by ATAC-seq, H3K4me1, and H3K27ac ChIP-seq (Fig. 3c). Importantly, 4C chromatin conformation capture analysis showed that e4 spatially interacted with the *MYCN* promoter on the amplicon (Fig. 3c). Thus, e4 presents as a functional enhancer and appears to contribute to *MYCN* expression, even in the context of class I *MYCN* amplification.

### Enhancer hijacking compensates for local enhancer loss

In contrast to class I amplicons, class II amplicons lacked key local enhancers and nevertheless expressed relatively high levels of *MYCN* per gene copy, raising the possibility of alternative routes of *MYCN* regulation (Supplementary Fig. 7). The lack of a strong local regulatory element on class II amplicons and our observation of frequent co-amplification of distal SE (Fig. 2c) led us to hypothesize that ectopic enhancers might be recruited to enable *MYCN* expression in class II amplicons. In agreement with our hypothesis, primary neuroblastomas with class II amplicons were more likely to harbor complex amplifications containing more than one amplified fragment in the genome (66.7% vs. 35.7%, Fisher’s exact test *P* = 0.003; Fig. 3e). In this largely array-based dataset, we cannot exclude fragments that are not structurally fused to the *MYCN* locus. However, it is unlikely that highly amplified loci have very similar copy number if they are not part of a common amplicon. We therefore filtered for fragments with highly similar copy number as *MYCN* (log ratio difference ≤0.1) and again found increased amplicon complexity for class II (class II 36.0% vs. class I 11.6%, Fisher’s exact test *P* = 0.003). All but one class II amplicon co-amplified at least one CRC-driven enhancer element distal of *MYCN*. Some of these enhancers were recurrently found on class II amplicons, including an enhancer 1.2 Mb downstream of *MYCN* that was co-amplified in 20.8% (5/24) of *MYCN*-amplified neuroblastomas, 2.1-fold higher than expected for randomized amplicons that include *MYCN* but not e4 (Fig. 3f). Thus, class II *MYCN* amplicons are characterized by high structural complexity, allowing for the replacement of local enhancers through hijacking of distal CRC-driven enhancers.

**Fig. 4:**
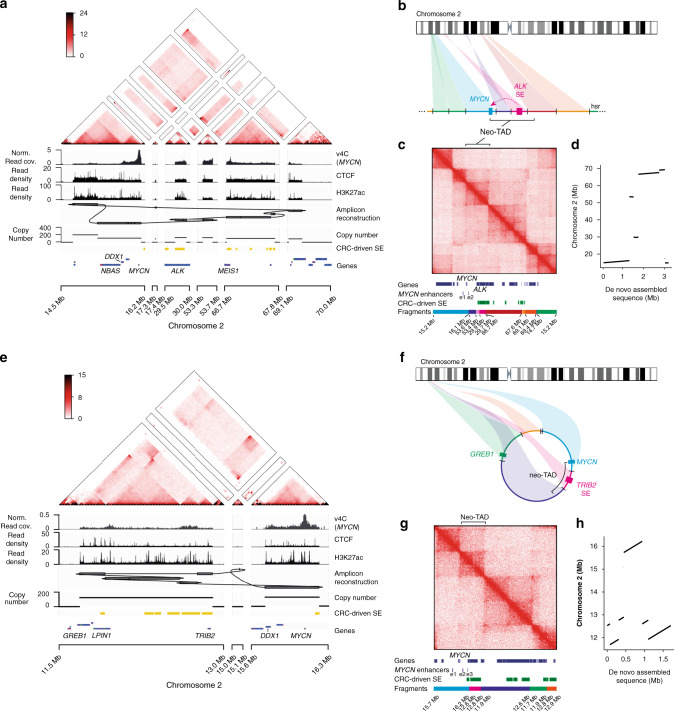
Reconstruction and epigenetic markup of class II *MYCN* amplicons. **a**, **e** Short-read-based reconstruction and epigenomic characterization of the *MYCN* amplicon in IMR-5/75 (**a**) and CHP-212 (**e**) cells. Top to bottom: Hi-C map (color indicating Knight–Ruiz normalized read counts in 25 kb bins), virtual 4C (*MYCN* viewpoint, v4C), CTCF ChIP-seq, H3K27Ac ChIP-seq, Amplicon reconstruction, copy-number profile, super-enhancer locations (yellow), gene positions (blue). **b**, **f** Schematic representation of the class II amplicon described in **a**, **e**, showing ectopic enhancers and insulator reshuffling leading to locally disrupted regulatory neighborhoods on the HSR in IMR-5/75 (**b**) and on ecDNA in CHP-212 (**f**). **c**, **g** Alignment of Hi-C reads to the reconstructed *MYCN* amplicon in IMR-5/75 (**c**) and CHP-212 (**g**) and positions of genes, local *MYCN* enhancers and CRC-driven super-enhancers on the amplicon. **d**, **h** Mapping of the long-read sequencing-based de novo assembly of the *MYCN* amplicon in IMR-5/75 (**d**) and CHP-212 (**h**) on chromosome 2. Source data are provided as a Source Data file.

To determine the structure and epigenetic regulation of class II amplicons in detail, we inspected long-read-based de novo assemblies and short-read-based reconstructions of IMR-5/75 and CHP-212 *MYCN* amplicons. High-throughput chromosome conformation capture (Hi-C) was performed and validated the reconstructions, recapitulating the order and orientation of the joined fragments. IMR-5/75 was characterized by a linear HSR class II *MYCN* amplicon, not including e3–e5 (Fig. 3b). Inspection of the IMR-5/75 *MYCN* amplicon structure revealed that the amplicon consisted of six distant genomic regions, which were joined together to form a large and complex chimeric amplicon (Fig. 4a–d). One of the fragments was likely included as a tandem duplication on the amplicon (Supplementary Fig. 8a). In line with enhancer hijacking, a segment of *ALK* containing a large SE, marked by H3K27ac and chromatin accessibility as measured using ATAC-seq, was juxtaposed with *MYCN* on the chimeric amplicon. Similar to e4, this enhancer was bound by adrenergic CRC factors in non-amplified cells (Supplementary Fig. 9a). In CHP-212, *MYCN* is amplified on ecDNA, as confirmed by fluorescence in situ hybridization (Supplementary Fig. 10). Both de novo assembly and short-read-based reconstruction of the amplicon confirmed the circular *MYCN* amplicon structure independently (Fig. 4f–h). Similar to IMR-5/75, distal fragments containing CRC-driven SEs were joined to the *MYCN* neighborhood (Fig. 4e, f and Supplementary Fig. 9b).

### Neo-topologically associated domains (TADs) form on chimeric *MYCN* amplicons

To analyze the three-dimensional conformation of circular and linear amplicons we mapped Hi-C reads to the reconstructed amplicon (Fig. 4c, g). Notably, high-frequency interactions in the corners of the maps opposite to the main diagonal confirmed the circularity of CHP-212 amplicon and tandem duplication-type amplification in IMR-5/75. On a more local level, Hi-C can be used to characterize TADs, i.e. regions of increased spatial interaction which contribute to gene control and arise through chromatin loops anchored at CTCF-marked insulator elements^32^. In IMR-5/75 and CHP-212, we observed insulated TADs as in the rest of the genome, suggesting that general rules of chromatin topology are retained on ecDNA and HSRs. Due to the rearrangements in CHP-212, the *MYCN* gene became part of a new chromatin domain (neo-TAD) where genes, enhancers, and insulators from distal parts of the genome form a new spatially interacting neighborhood. *MYCN* itself was located at the intersection of two smaller sub-TADs. The first sub-TAD originated from the wild-type genome as an intact unit. The second sub-TAD resulted from the fusion of the *MYCN* locus with another region from a distal part of chromosome 2 (chr2:12.6–12.8 Mb) containing CRC-driven SEs (Fig. 4g and Supplementary Fig. 9b). The fused segments were part of one TAD and not separated by a boundary, which enables the interaction of *MYCN* with the ectopic SEs. A similar situation was observed for the linear amplicon in IMR-5/75, where frequent contacts between *MYCN* and SEs from the genomic regions juxtaposed to *MYCN*, containing intronic parts of *ALK*, were detected using Hi-C (Fig. 4c and Supplementary Figs. 8b and  9a). Notably, hijacked SEs covered 46% and 44% of the neo-TAD for IMR-5/75 and CHP-212, respectively. In both cell lines, additional fragments of chromosome 2 were fused to the SE-containing region. These contained neo-TAD boundaries as determined by Hi-C (Fig. 4d, g). All neo-TAD boundaries were marked by CTCF ChIP-seq peaks, with canonical forward–reverse motif orientations in IMR-5/75 (Supplementary Fig. 9a). In CHP-212, no unambiguous CTCF motif orientations at the downstream neo-TAD border were identified (Supplementary Fig. 9b). In both cases, however, the new insulators originated from genomic locations other than the *MYCN* fragment and the SE-containing fragments. In addition to the observed TAD structures, weaker off-diagonal interactions were visible, suggesting a heterogeneous group of structurally different variants of the original amplicon. Nevertheless, the TAD structure, boundaries, and loops were clearly visible on the reconstructed Hi-C map (Fig. 4c). Thus, hijacking of ectopic enhancers and insulators can compensate for the loss of endogenous regulatory elements on intra- and extrachromosomal circular class II *MYCN* amplicons via the formation of neo-TADs, which may explain the higher structural complexity of *MYCN* amplicons lacking endogenous enhancers.

### Nanopore sequencing characterizes amplicon methylation

**Fig. 5:**
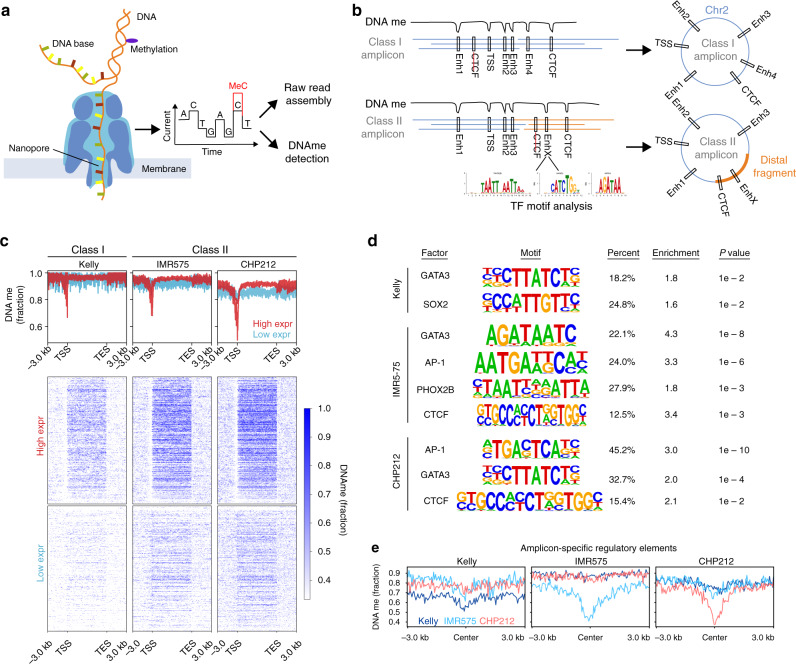
Nanopore sequencing characterizes DNA methylation on *MYCN* amplicons. **a** Schematic of experimental approach. **b** Schematic representation of how nanopore sequencing facilitates de novo amplicon assembly and can be used to simultaneously to detect regulatory elements through DNA methylation analysis. **c** Composite DNA methylation signal detected using nanopore sequencing over genes expressed at high (HighExpr) vs. low levels (LowExpr). **d** Motif analysis based on accessibility in regulatory elements co-amplified on *MYCN* amplicons (unadjusted *P* values from one-sided binomial test against nucleotide composition-matched background sequences). **e** Amplicon-specific methylation pattern detected in three neuroblastoma cell lines (Kelly, IMR-5/75, CHP-212) using nanopore sequencing-based DNA methylation analysis.

In addition to allowing the alignment-free de novo assembly of the *MYCN* amplicon in several samples (Fig. 4b–d, f–h and Supplementary Figs. 5 and 6), nanopore sequencing also allows for the direct measurement of DNA methylation without the need for bisulfite conversion (Fig. 5a)^33^. While DNA methylation at regulatory elements is often associated with repression, a trough in DNA methylation may indicate a transcription factor-binding event, a poised or active gene-regulatory element, or a CTCF-occupied insulator element (Fig. 5b). In theory, nanopore sequencing and assembly might allow for the simultaneous inference of both structure and regulatory landscape (Fig. 5b). Prior to evaluating the *MYCN* amplicons, the DNA methylation landscape of highly expressed and inactive genes demonstrated the expected distribution of decreased methylation at active promoters and increased methylation within active gene bodies (Fig. 5c). In order to assess the DNA methylation status of putative regulatory elements near *MYCN*, we first used the amplicon-enriched ATAC-seq peaks to classify relevant motif signatures (Fig. 5d). While *MYCN* was surrounded by the expected CRC-driven regulatory elements at the overlapping core enhancers as well as some CTCF sites, both their number and location varied, indicating sample-specific sites of regulation. Indeed, DNA methylation decreased in accordance with sites specific to a given sample (Fig. 5e), opening up the possibility of using these data to infer regulatory elements in patient samples, when no orthogonal epigenomic data are available.

### Class II amplicons clinically phenocopy class I amplicons

**Fig. 6:**
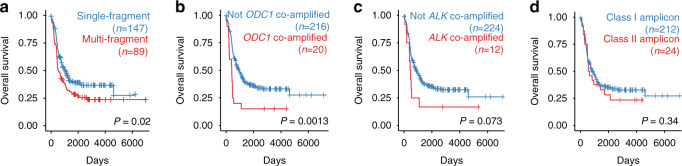
Class II amplicons clinically phenocopy class I amplicons. Kaplan Meier survival analysis of patients with *MYCN*-amplified neuroblastoma, comparing single-fragment vs. multi-fragment amplification (**a**), co-amplification of *ODC1* vs. no co-amplification (**b**), co-amplification of *ALK* vs. no co-amplification (**c**), and class I amplicons vs. class II amplicons (**d**; *N* = 236 *MYCN*-amplified neuroblastomas; *P* value based on two-sided log-rank test). Source data are provided as a Source Data file.

*MYCN*-amplified neuroblastoma is characterized by significant clinical heterogeneity, which cannot entirely be explained by genetic differences. Whether the structure of the *MYCN* amplicon itself could account for some of this variation is currently unknown. In line with previous reports^31^, higher counts of amplified fragments were associated with a more malignant clinical phenotype (Fig. 6a). Co-amplification of *ODC1*, a gene located 5.5 Mb upstream of *MYCN* and co-amplified in 9% (21/240) of *MYCN*-amplified neuroblastomas (Fig. 2c), defined an ultra-high-risk genetic subgroup of *MYCN*-amplified neuroblastoma (hazard ratio (HR) 2.3 (1.4–3.7), log-rank test *P* = 0.001; Fig. 6b). Similarly, *ALK* co-amplification, present in 5% (12/240) of *MYCN*-amplified tumors, was also associated with adverse clinical outcome (HR 1.8 (0.94–3.4), log-rank test *P* = 0.073; Fig. 6c). In contrast, differences in the *MYCN* amplicon enhancer structure, i.e. class I vs. class II amplification, did not confer prognostic differences (HR 1.3 (0.78–2.1), log-rank test *P* = 0.34; Fig. 6d). We therefore conclude that chimeric co-amplification of proto-oncogenes partly explains the malignant phenotype of neuroblastomas with complex *MYCN* amplicons, whereas enhancer hijacking in class II amplicons does not change clinical behavior, fully phenocopying class I *MYCN* amplicons.

## Discussion

**Fig. 7:**
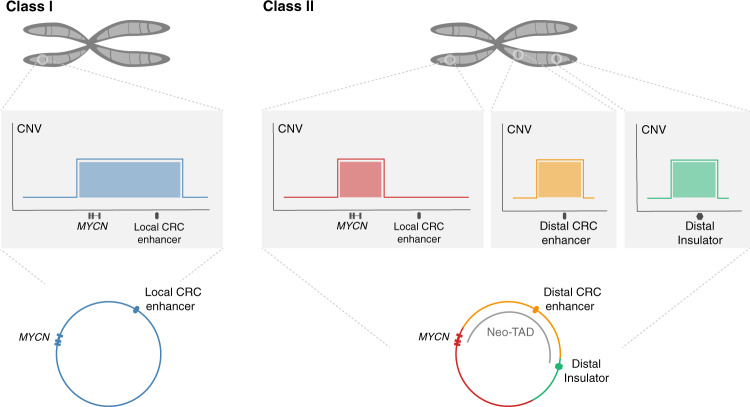
Enhancer co-amplification determines *MYCN* amplicon patterns. In most cases, *MYCN* and its local gene-regulatory neighborhood including a CRC-driven super-enhancer is amplified (Class I). If the local neighborhood is not co-amplified, amplicons are more complex and recruit distal gene-regulatory elements (Class II).

Here, we show that neuroblastoma-specific CRC-driven enhancers contribute to *MYCN* amplicon structure in neuroblastoma and retain the classic features of active enhancers after genomic amplification. While most *MYCN* amplicons contain local enhancers, ectopic enhancers are regularly incorporated into chimeric amplicons lacking local enhancers, leading to enhancer hijacking (Fig. 7).

A large subset of neuroblastomas was recently found to be driven by a small set of transcription factors that form a self-sustaining CRC, defined by their high expression and presence of super-enhancers^15–18^. The extent to which *MYCN* itself is directly regulated by CRC factors was previously unclear, complicated by the challenge of interpreting epigenomic data on amplicons^16^. Our results provide empiric evidence that *MYCN* is driven by CRC factors, even in the context of *MYCN* amplification. This could mechanistically explain the previous observation that genetic depletion of CRC factors represses *MYCN* expression even in *MYCN*-amplified cells^16^. The finding that ectopic enhancers driven by the CRC are juxtaposed to *MYCN* on amplicons that lack local enhancers further strengthens the relevance of the CRC in *MYCN* regulation.

In line with our observation of local enhancer co-amplification, Morton et al.^19^ recently described that local enhancers are significantly co-amplified with other proto-oncogenes in other cancer entities. They showed that experimentally interfering with local *EGFR* enhancers in *EGFR*-amplified glioblastoma impaired oncogene expression and cell viability in *EGFR*-amplified as well as non-amplified cases. Consistent with our findings, the authors identified a region overlapping e4 that was significantly co-amplified in *MYCN*-amplified neuroblastomas, corresponding to class I amplicons observed in our cohort. In contrast to Morton et al.^19^, who suggest that the inclusion of local enhancers is necessary for proto-oncogene expression on amplicons, we show that exceptions to this rule occur in a significant subset of *MYCN-*amplified neuroblastomas. In such cases, amplicons characterized by highly complex chimeric structure enable the reshuffling of ectopic enhancers and insulators to form neo-TADs that can compensate for disrupted local neighborhoods through enhancer hijacking.

More generally, we show that TADs also form on ecDNA, in parallel with recent findings by Wu et al.^34^. We extend this observation to HSRs, which form extremely expanded stretches of chromatin in interphase nuclei and lose chromosomal territoriality^35^. Gene activation by enhancer adoption requires the fusion of distant DNA fragments and the formation of new chromatin domains, called neo-TADs^36^. In some cases, this fusion requires a convergent directionality of CTCF sites in order to form a new boundary and drive aberrant gene expression^37^. This has been explained by a model of blocked loop extrusion at forward–reverse oriented CTCF sites^32^. We found convergent CTCF for the neo-TAD in IMR-5/75 but not necessarily for the one in CHP-212. However, non-convergent CTCF sites have been consistently reported before and characterize at least one in ten CTCF-mediated chromatin loops in the wild-type genome^38,39^. Although the exact underpinnings are not yet clear, CTCF convergence is likely not required in some genomic contexts, which could be the case in CHP-212 and other ecDNA amplicons.

Reconstruction of amplicons has previously relied on combining structural breakpoint coordinates to infer the underlying structure. This regularly resulted in ambiguous amplicon reconstructions, which had to be addressed by secondary data such as chromium linked reads or optical mapping^4,6,34^. We demonstrate the feasibility of long-read de novo assembly for the reconstruction of amplified genomic neighborhoods. De novo assembly was able to reconstruct entire ecDNA molecules and confirm the tandem duplicating nature of HSRs. Integrating de novo assembly with methylation data from nanopore sequencing reads will likely benefit further studies of other proto-oncogene-containing amplicons by enabling the characterization of the interplay between structure and regulation in highly rearranged cancer genomes.

Functional studies have shown that both *ODC1* and *ALK* are highly relevant in neuroblastoma^40,41^. Co-amplification with *MYCN* has been reported before^31^, but to our knowledge the clinical relevance of co-amplification had not been determined so far. Similar to our previous observations of *PTP4A2* co-amplification on chimeric ecDNA^10^, we demonstrate here that proto-oncogenes reside side-by-side on the same ecDNAs, sometimes even sharing the same regulatory neighborhood. It is tempting to speculate that this structural coupling of genes could confer MYCN-independent but *MYCN*-amplicon-specific, collateral therapeutic vulnerabilities in *MYCN*-amplified tumors.

We conclude that the structure of genomic amplifications can be explained by a selective pressure to amplify oncogenes together with suitable non-coding regulatory elements. CRC-driven enhancers are required for successful *MYCN* amplification and remain functional throughout this process. Even though the majority of amplicons contain endogenous enhancers, these can be functionally replaced by ectopic CRC-driven enhancers that are juxtaposed to the oncogene through complex chimeric amplicon formation. We envision that our findings also extend to oncogene amplifications in other cancers and will help identify functionally relevant loci among the diverse array of complex aberrations that drive cancer.

## Methods

### Cell lines

Neuroblastoma cell lines were a gift from F. Speleman (Cancer Research Institute Ghent, Ghent, Belgium; NGP), F. Westermann (German Cancer Research Center, Heidelberg, Germany; IMR-5/75), obtained from the German Collection of Microorganisms and Cell Cultures (DSMZ GmbH, Braunschweig, Germany; Kelly), or obtained from the American Type Culture Collection (ATCC, Manassas, VA; CHP-212). Cell line identity was verified by STR genotyping (Genetica DNA Laboratories, Burlington, NC and IDEXX BioResearch, Westbrook, ME) and absence of Mycoplasma sp. contamination was determined with a Lonza MycoAlert system (Lonza Group Ltd, Basel, CH). All cell lines were cultured in RPMI-1640 medium (Thermo Fisher Scientific, Inc., Waltham, MA) with 1% Penicillin/Streptomycin and 10% FCS.

### RNA-seq

Public RNA-seq data were downloaded from Gene Expression Omnibus (GSE90683)^15^. FASTQ files were quality controlled (FASTQC 0.11.8) and adapters were trimmed (BBMap 38.58). We mapped reads to GRCh37 (STAR 2.7.1 (ref. ^42^) with default parameters), counted them per gene (Ensembl release 75, featureCounts from Subread package 1.6.4 (ref. ^43^)), and normalized for library size and composition (sizeFactors from DESeq2 1.22.2 (ref. ^44^)).

### ChIP-seq

For the cell lines CHP-212, NGP, and Kelly, 5–10 × 10^6^ cells were digested with Trypsin–EDTA 0.05% (Gibco) for 10 min at 37 °C. The cells were mixed with 10% FCS–PBS, and a single-cell suspension was obtained using a 40-µm cell strainer. After centrifugation, cells were resuspended in 10% FCS–PBS again and fixed in 1% paraformaldehyde (PFA) for 10 min at room temperature. The reaction was quenched with 2.5 M glycine (Merck) on ice and centrifuged at 400*g* for 8 min. We resuspended cell pellets in lysis buffer (50 mM Tris, pH 7.5; 150 mM NaCl; 5 mM EDTA; 0.5% NP-40; 1.15% Triton X-100; protease inhibitors (Roche), 5 mM Na-butarate), and nuclei were pelleted again by centrifugation at 750*g* for 5 min. For sonication, nuclei were resuspended in sonication buffer (10 mM Tris–HCl, pH 8.0; 100 mM NaCl; 1 mM EDTA; 0.5 mM EGTA; 0.1% Na-deoxycholate; 0.5% *N*-lauroylsarcosine; protease inhibitors (Roche complete), 5 mM Na-butarate). Chromatin was sheared using a Diagenode Bioruptor (35–40 cycles with a 30 s on/off pulse and HI power mode) until reaching a fragment size of 200–500 base pairs (bp). Lysates were clarified from sonicated nuclei, and protein–DNA complexes were immunoprecipitated overnight at 4 °C with the respective antibody. A total of 10–15 μg chromatin was used for each replicate of histone ChIP and 20–25 µg of transcription factor ChIP. For the immunoprecipitation^45^ in 1200 µl precipitation buffer (10 mM Tris–HCl, pH 8.0; 100 mM NaCl; 1 mM EDTA; 0.5 mM EGTA; 0.1% Na-deoxycholate; 0.5% *N*-lauroylsarcosine; protease inhibitors (Roche complete), 5 mM Na-butarate, 1% Triton X-100), Anti-H3K27ac (Diagenode c15410174; lot A7071-001P; dilution 1:500), anti-H3K4m1 (Abcam; ab8895; lot GR141677-1; dilution 1:1200), anti-RAD21 (Abcam; ab992; lot GR221348-8; dilution 1:150) and anti-CTCF (Active Motif; 613111; lot 34614003; dilution 1:150) antibodies were used. Sequencing libraries were prepared using standard Nextera adapters (Illumina) according to the supplier’s recommendations. Twenty-five million reads per sample were sequenced on a HiSeq 4000 sequencer (Illumina) in 75 bp single read mode.

Additional public ChIP-seq FASTQ files were downloaded from Gene Expression Omnibus (GSE18927, GSE90683, GSE24447, and GSE28874)^15,46^ and from ArrayExpress (E-MTAB-6570)^17^. FASTQ files were quality controlled (FASTQC 0.11.8) and adapters were trimmed (BBMap 38.58). Reads were then aligned to hg19 (BWA-MEM 0.7.15 (ref. ^47^) with default parameters) and duplicate reads removed (Picard 2.20.4). We generated BigWig tracks by extending reads to 200 bp for single-end libraries and extending to fragment size for paired-end libraries, filtering by ENCODE DAC blacklist and normalizing to counts per million in 10 bp bins (deepTools 3.3.0 (ref. ^48^)). Peaks were called using MACS2 (2.1.2)^49^ with default parameters. Super-enhancers were called for H3K27ac data using LILY^15^ (https://github.com/BoevaLab/LILY) with default parameters. ChIP-seq data were quality controlled using RSC and NSC (Phantompeakqualtools 1.2.1). CTCF motifs within CTCF ChIP-seq peaks were identified using JASPAR2018 (ref. ^50^) and the TFBSTools (1.20.0)^51^ function matchPWM with min.score = “75%”. Copy-number ratio was estimated by binning ChIP-seq input reads (primary alignments of mapping quality 20 or higher) in 1 kb bins, correcting for GC content, normalization, and segmentation using QDNAseq (1.22.0)^52^.

### ATAC-seq

ATAC-seq samples were processed as reported in Buenrostro et al.^53^ with some adaptations: After a treatment of 5–10 × 10^6^ cells with Trypsin–EDTA 0.05% (Gibco) for 10 min at 37 °C, a 40-µm cell strainer was used to obtain a single-cell suspension. 5 × 10^5^ cells were washed with cold 1× PBS and lysed with freshly prepared lysis buffer (10 mM Tris-Cl pH 7.4, 10 mM NaCl, 3 mM MgCl_2_, 0.1% (v/v) Igepal CA-630) by pipetting six times up and down and a subsequent incubation on ice for 1 min. After a centrifugation at 500*g* for 5 min at 4 °C the pellet was resuspended with gentle mixing in transposition reaction mix (25 µl 2× TD, 2.5 µl TDE1 and 22.5 µl H_2_O, Illumina). Immediately after the transposition reaction, the DNA was purified using a MinElute PCR Purification Kit (Qiagen). The transposed DNA was amplified using a Nextera PCR Kit (Illumina) according to the supplier’s recommendation. The maximum number of cycles was determined with qPCR to reduce PCR bias. For sequencing, libraries were generated using Illumina/Nextera adapters and size selected (100–1000 bp) with AMPure Beads (Beckman Coulter). Approximately 100 million 75 bp paired-end reads were acquired per sample on the HiSeq 4000 system (Illumina). Additional public ATAC-seq FASTQ files were downloaded from Gene Expression Omnibus (GSE80154)^54^. Adapter trimming, alignment, and duplicate removal as for ChIP-seq. We generated BigWig tracks by extending paired-end reads to fragment size, filtering by the ENCODE DAC blacklist and normalizing to counts per million in 10 bp bins (deepTools 3.3.0 (ref. ^48^)). Peaks were called using MACS2 (2.1.2)^49^ with default parameters.

### Hi-C

3C libraries for Hi-C and 4C were prepared from confluent neuroblastoma cells according to the cell culture section above. Hi-C experiments were performed as duplicates. 5–10 × 10^6^ cells were washed twice with PBS and digested with Trypsin–EDTA 0.05% (Gibco) for 10 min at 37 °C. A 40-µm cell strainer was used to obtain single cells. The cell suspension was pelleted at 300*g* for 5 min and resuspended with cold 10% FCS. Subsequently, the cells were fixed by adding an equal volume of 4% formaldehyde (Sigma-Aldrich). The suspension was mixed for 10 min while shaking at room temperature in 50 ml tubes. Exactly after 10 min the fixation was quenched with 500 µl 1.425 M glycine (Merck) on ice. The suspension was pelleted at 400*g* for 8 min and resuspended in cold lysis buffer (50 mM Tris, pH 7.5; 150 mM NaCl; 5 mM EDTA; 0.5% NP-40; 1.15% Triton X-100; protease inhibitors (Roche)). After a washing step with cold 1× PBS and centrifugation at 750*g* for 5 min, the pellet was washed with 1× DpnII buffer (NEB) and resuspended in 50 µl 0.5% SDS and incubated for 10 min at 62 °C. After that 145 µl water and 25 µl 10% Triton (Sigma) was added to quench the SDS followed by a incubation at 37 °C for 30 min. For the restriction enzyme digestion, 25 µl DpnII buffer and 100 U DpnII was added. The digestion reaction was incubated for 2 h at 37 °C, after 1 h another 10 U were added and then heat inactivated at 65 °C for 20 min.

The digested sticky ends were filled up with 10 mM dNTPs (without dATP) and 0.4 mM biotin-14-dATP (Life Technologies) and 40 U DNA Pol I, Large Klenow (NEB) at 37 °C for 90 min. Biotinylated blunt ends were then ligated using a ligation reaction (663 µl water, 120 µl 10× NEB T4 DNA ligase buffer (NEB), 100 µl 10% Triton X-100 (Sigma), 12 µl 10 mg/ml BSA, and 2400 U of T4 DNA ligase (NEB)) overnight at 16 °C with slow rotation.

For the 3C library preparation, DNA was sheared using a Covaris sonicator (duty cycle: 10%; intensity: 5; cycles per burst: 200; time: six cycles of 60 s each; set mode: frequency sweeping; temperature: 4–7 °C). After sonication, religated DNA was pulled down using 150 µl of 10 mg/ml Dynabeads Streptavidin T1 beads (Thermo Fisher) according to the supplier’s recommendation. Sheared and pulled down DNA was treated using a 100 µl end-repair reaction (25 mM dNTPs, 50 U NEB PNK T4 Enzyme, 12 U NEB T4 DNA polymerase, 5 U NEB DNA pol I, Large (Klenow) Fragment, 10× NEB T4 DNA ligase buffer with 10 mM ATP) and incubated for 30 min at 37 °C.

Universal sequencing adaptor were added using the NEBnext Ultra DNA Library Kit (NEB) according to the supplier’s recommendation. The PCR cycle number was adjusted to 4–12 based on the initial DNA concentration. The final libraries were purified using AMPure Beads (Beckman Coulter) and samples were sequenced with Ilumina Hi-Seq technology according to the standard protocols and 75 bp (shallow CHP-212 Hi-C, deep IMR-5/75) and 150 bp (shallow IMR-5/75 Hi-C) paired-end mode. Around 100 million reads were generated per IMR-5/75 replicate (deep IMR-5/75 Hi-C) and around 5–25 million reads per replicate were generated for shallow CHP-212 and shallow IMR-5/75 Hi-C.

FASTQ files were processed using the Juicer pipeline v1.5.6, CPU version^55^, which was set up with BWA v0.7.17 (ref. ^47^) to map short reads to reference genome hg19, from which haplotype sequences were removed and to which the sequence of Epstein–Stein–Barr Virus (NC_007605.1) was added. Replicates were processed individually. Mapped and filtered reads were merged afterwards. A threshold of MAPQ ≥ 30 was applied for the generation of Hi-C maps with Juicer tools v1.7.5 (ref. ^55^). Knight–Ruiz normalization was used for Hi-C maps^38,56^. In cases with copy-number variation within the amplicon, we visually compared unnormalized, Knight–Ruiz-normalized and local iterative correction-normalized^57^ maps to confirm the robustness of our conclusions across different normalization approaches (Supplementary Fig. 8). Virtual 4C signal for the *MYCN* locus was generated by the mean Knight–Ruiz-normalized Hi-C signal across three 5 kb bins (chr2: 16,075,000–16,090,000).

### 4C-seq

For 4C-seq libraries, a starting material of 5 × 10^6^–1 × 10^7^ cells were used. The fixation and lysis were performed as described in the “Hi-C” section. After the first digestion with DpnII (NEB), sticky ends were religated in a 50 ml falcon tube (700 µl 10 ligation buffer (Fermentas), 7 ml H_2_O, 50 U T4 DNA ligase (Thermo); overnight at 16 °C) and DNA de-cross linked and cleaned as described in the “HiC” section. Subsequently, a second digestion (150 µl sample, 50 µl 10× Csp6I buffer (Thermo), 60 U Csp6I (Thermo) 295 µl H_2_O; overnight at 37 °C) and another re-ligation was performed. For the *MYCN* promoter viewpoint, DNA was purified using a PCR clean up Kit (Qiagen) and 1.6 µg DNA was amplified by PCR (Primer 1 5′-GCAGAATCGCCTCCG-3′, Primer 2 5′-CCTGGCTCTGCTTCCTAG-3′). For the library reaction, primers were modified with TruSeq adapters (Illumina): Adapter1 5′-CTACACGACGCTCTTCCGATCT-3′ and Adapter2 5′-CAGACGTGTGCTCTTCCGATCT-3′. The input of a single 4C PCR reaction was between 50 and 200 ng depending on the complexity. The reaction was performed in a 50 µl volume using the Expand Long Template System (Roche) and 29 reaction cycles. After the PCR all reactions were combined and the DNA purified with a PCR clean up Kit (Qiagen). All samples were sequenced with the HiSeq 4000 (Illumina) technology according to the standard protocols and with around 20 million single-end reads per sample.

Reads were pre-processed, filtered for artefacts, and mapped to the reference genome GRCh37 using BWA-MEM as described earlier^36^. After removing the viewpoint fragment as well as 1.5 kb up- and downstream of the viewpoint the raw read counts were normalized per million mapped reads (RPM) and a window of 10 fragments was chosen to smooth the profile.

### Whole-genome sequencing

Cells were harvested and DNA was extracted using the NucleoSpin Tissue kit (Macherey-Nagel GmbH & Co. KG, Düren, Germany). Libraries for whole-genome sequencing were prepared with the NEBNext Ultra II FS DNA Library Prep Kit for Illumina (New England BioLabs, Inc., Ipswich, MA). Libraries were sequenced on a MGISEQ-2000 (NGP; MGI Tech Co. Ltd, Shenzhen, China), HiSeq X (IMR-5/75, Kelly; Illumina, Inc., San Diego, CA), and NovaSeq 6000 (CHP-212; Illumina, Inc., San Diego, CA) with 2 × 150 bp paired-end reads. Quality control, adapter trimming, alignment, duplicate removal as for ChIP-seq data. Copy-number variation was called (Control-FREEC^58^ 11.4 with default parameters). Structural variants were called using SvABA^59^ (1.1.1) in germline mode and discarding regions in a blacklist provided by SvABA (https://data.broadinstitute.org/snowman/svaba_exclusions.bed).

### Nanopore sequencing

Cells were harvested and high molecular weight DNA was extracted using the MagAttract HMW DNA Kit (Qiagen N.V., Venlo, Netherlands). Size selection was performed to remove fragments <10 kilobases (kb) using the Circulomics SRE kit (Circulomics Inc., Baltimore, MD). DNA content was measured with a Qubit 3.0 Fluorometer (Thermo Fisher) and sample quality control was performed using a 4200 TapeStation System (Agilent Technologies, Inc., Santa Clara, CA). Libraries were prepared using the Ligation Sequencing Kit (SQK-LSK109, Oxford Nanopore Technologies Ltd, Oxford, UK) and sequenced on a R9.4.1 MinION flowcell (FLO-MIN106, Oxford Nanopore Technologies Ltd, Oxford, UK). Quality control was performed using NanoPlot 1.0.0 (ref. ^60^). For the NGP cell line, DNA was extracted with the NucleoSpin Tissue kit (Macherey-Nagel GmbH & Co. KG, Düren, Germany) and libraries were prepared using the ONT Rapid Kit (SQK-RBK004, Oxford Nanopore Technologies Ltd, Oxford, UK). Guppy 2.3.7 (Oxford Nanopore Technologies Ltd, Oxford, UK) was used for basecalling with default parameters. For de novo assembly, Flye 2.4.2 (ref. ^61^) was run in metagenomics assembly mode on the unfiltered FASTQ files with an estimated genome size of 1 Gb. Contigs were mapped back to hg19 using minimap2 2.16 (ref. ^62^) with parameter -ax asm5. Assembly results were visualized with Bandage 0.8.1 (ref. ^63^) and Ribbon 1.0 (ref. ^64^). CpG methylation was called from the unfiltered raw FAST5 files using Megalodon 0.1.0 (Oxford Nanopore Technologies Ltd, Oxford, UK). Motif signatures were derived with Homer (4.9.1)^65^ using the binomial test against nucleotide composition-matched background sequences. CpG methylation composite profiles were created by averaging signal in 50 bp bins using computeMatrix in deepTools (3.3.0)^48^.

### Fluorescence in situ hybridization

Cells were grown to 200,000 per well in six-well plates and metaphase-arrested using Colcemid (20 µl/2 ml; Roche #10295892001) for 30 min–3 h, trypsinized, centrifuged (200*g*/10 min), washed, and pelleted. Five milliliters of 0.4% KCl (4 °C; Roth #6781.1) was added to the pellet and incubated for 10 min. One milliliter KCl and 1 ml MeOH/acetic acid 3:1 (Roth #4627.2, #KK62.1) was added drop-wise. In all, 2/5/5 ml of MeOH/acetic acid was added in between centrifugation steps (200*g*/10 min), respectively. Suspension was dropped on a slide from a height of 40 cm. Slides were washed with PBS (Gibco, #70011036) and digested for 10 min in 0.04% pepsin solution in 0.001 N HCl. Slides were washed in 0.5× SSC, dehydrated with 70%/80%/100% EtOH (3 min each), and air-dried. Ten microliters of the probe (Vysis LSI N-MYC; #07J72-001; Lot #472123; Abbott Laboratories, Abbott Park, IL) were added and coverslips fixed on the slide. Slides were incubated at 75 °C for 10 min and at 37 °C overnight. The coverslip was removed and the slide was washed in 0.4× SSC/0.3% IGEPAL (CA-630, #18896; Sigma-Aldrich Inc.) for 3 min at 60 °C and 2× SSC/0.1% IGEPAL for 3 min at RT. Five microliters DAPI (Vectashield, #H-1200, Vector) was added. A coverslip was added and fixed with nail polish.

### Enhancer calling

*MYCN*-expressing cell lines were defined as cell lines with size-Factor normalized expression of 100 or above based. We identified enhancer candidate regions in a ±500 kb window around *MYCN*. We focused on regions with a H3K27ac peak in the majority of *MYCN-*expressing, non-*MYCN-*amplified cell lines, i.e. three or more. If the gap between two such regions was less than 2 kb, they were joined. These regions were then ranked by the maximum difference in H3K27ac signal fold change between non-amplified, *MYCN*-expressing, and non-expressing cell lines. We chose the five highest-ranking regions as candidate regulatory elements. Enhancer regions were screened for transcription factor-binding sequences from the JASPAR2018 (ref. ^50^) and JASPAR2020 (ref. ^66^) database using the TFBSTools 1.20.0 (ref. ^51^) function matchPWM with min.score = “85%”. CRC-driven super-enhancers were defined as all regions with a LILY-defined super-enhancer in *MYCN*-expressing, non-*MYCN*-amplified cell lines that overlapped with a GATA3, HAND2, or PHOX2B peak in CLB-GA.

### Analysis of neuroblastoma copy-number data

Public data were downloaded from https://github.com/padpuydt/copynumber_HR_NB/ (ref. ^27^). Samples that were described as *MYCN*-amplified in the metadata but did not show *MYCN* amplification in the copy-number profile were excluded. In order to generate an aggregate copy-number profile, the genome was binned in 10 kb bins and number of samples with overlapping amplifications was counted per bin. Randomized copy-number profiles were generated by randomly sampling one of the original copy-number profiles on chromosome 2 and randomly shifting it such that *MYCN* is still fully included within an amplified segment. For class I-specific shuffling, e4 had to be included as well; for class II-specific shuffling, e4 was never included on the randomly shifted amplicon. Empirical *P* values for significant co-amplification were derived by creating 10,000 randomized datasets with each amplicon randomly shifted and comparing the observed co-amplification frequency to the distribution of co-amplification frequencies in the randomized data. Empirical *P* values were always one-sided and adjusted for multiple comparisons using the Benjamini–Hochberg procedure.

### Analysis of medulloblastoma copy number and ChIP-seq data

Medulloblastoma Affymetrix SNP6 data (10 cell lines, 1087 patient samples) were downloaded from Gene Expression Omnibus (GSE37385)^29^ and processed using rawcopy 1.1 (ref. ^67^) with default parameters. Segments with a log2 ratio ≥1.8 were classified as amplifications. The genome was binned in 10 kb bins and the number of samples with overlapping amplifications was counted per bin to generate composite copy-number plots.

Medulloblastoma H3K27ac ChIP-seq BigWig files and super-enhancer regions were downloaded from https://pecan.stjude.cloud/dataset/northcott (ref. ^30^). The medulloblastoma subgroup-wise average H3K27ac signal was computed in 1 kb bins.

### Amplicon reconstruction

All unfiltered SvABA structural variant calls were filtered to exclude regions from the ENCODE blacklist^68^ and small rearrangements of 1 kb or less. As we were only aiming at the rearrangements common to all amplicons, we only considered breakpoints with more than 50 variant-support reads (“allele depth”). gGnome^69^ was used to represent these data as a genome graph with nodes being breakpoint-free genomic intervals and edges being rearrangements (“alternate edge”) or connections in the reference genomes (“reference edge”). We considered only nodes with high copy number, i.e. with a mean whole-genome sequencing coverage of at least 10-fold the median coverage of chromosome 2. Then, reference edges were removed if its corresponding alternate edge was among the 25% highest allele-depth edges. The resulting graph was then searched for the circular, *MYCN*-containing walk that included the highest number of nodes without using any node twice. We used gTrack (https://github.com/mskilab/gTrack) for visualization. For custom Hi-C maps of reconstructed amplicon sequences of CHP-212 and IMR-5-75, respectively, the corresponding regions from chromosome 2 were copied, ordered, oriented, and compiled according to the results from the amplicon reconstruction and added to the reference genome. Additionally, these copied regions were masked with “N” at the original locations on chromosome 2 to allow a proper mapping of reads to the amplicon sequence. The contribution of Hi-C di-tags from these regions on chromosome 2 to the amplicon Hi-C map is expected be minor, because the copy number of amplicons is much higher than the number of wild-type alleles.

### Reporting summary

Further information on research design is available in the Nature Research Reporting Summary linked to this article.

## Supplementary information

Supplementary Information

Reporting Summary

## Data Availability

Sequencing data generated for this study are available at the Sequence Read Archive under accession PRJNA622577. Copy-number data for high-risk neuroblastoma were downloaded from https://github.com/padpuydt/copynumber_HR_NB/ (ref. ^27^). Public data supporting the findings of this manuscript were downloaded from the Gene Expression Omnibus under accessions GSE90683, GSE80152, GSE24447, GSE37385, GSE18927, and GSE28874 and from ArrayExpress under accession E-MTAB-6570. Medulloblastoma ChIP-seq data were downloaded from https://pecan.stjude.cloud/dataset/northcott. BigWig und narrowPeak files can be downloaded from https://data.cyverse.org/dav-anon/iplant/home/konstantin/helmsaueretal/. An accompanying UCSC genome browser track hub is provided for ChIP-seq and ATAC-seq data visualization (https://de.cyverse.org/dl/d/27AA17DA-F24C-4BF4-904C-62B539A47DCC/hub.txt). All other data are available from the corresponding authors upon reasonable request. Source data are provided with this paper.

## Source data

Source Data

## Code availability

Code is available at https://github.com/henssenlab/MYCNAmplicon.
