# Supplementary material for: Enhancer hijacking determines extrachromosomal circular MYCN amplicon architecture in neuroblastoma
Source: Nat Commun. 2020 Nov 16;11:5823. doi: 10.1038/s41467-020-19452-y (PMC7669906; doi:10.1038/s41467-020-19452-y)
Supplement: Supplementary file 1 — Supplementary Information [file 41467_2020_19452_MOESM1_ESM.pdf]

Supplementary Information for Helmsauer et al. *"Enhancer hijacking determines extrachromosomal MYCN amplicon architecture in neuroblastoma"*

Supplementary Figures

- Supplementary Fig. 1. Enhancer profiling of the *MYCN* locus
- Supplementary Fig. 2. Amplification patterns across clinical and experimental covariates
- Supplementary Fig. 3. Super enhancer co-amplification confers different *MYCN* amplicon structures of medulloblastoma subgroups
- Supplementary Fig. 4. H3K27 acetylation on the *MYCN* amplicon
- Supplementary Fig. 5. Long-read sequencing enables de novo assembly of *MYCN* neighborhoods
- Supplementary Fig. 6. De novo assembly confirms co-amplification of *MYCN* and e4 in Kelly and NGP
- Supplementary Fig. 7. *MYCN* expression on two amplicon classes
- Supplementary Fig. 8. A tandem duplication on the *MYCN* amplicon in IMR-5/75
- Supplementary Fig. 9. Enhancer hijacking and neo-TAD formation on the *MYCN* amplicon
- Supplementary Fig. 10. *MYCN* fluorescence in situ hybridization in neuroblastoma cell lines

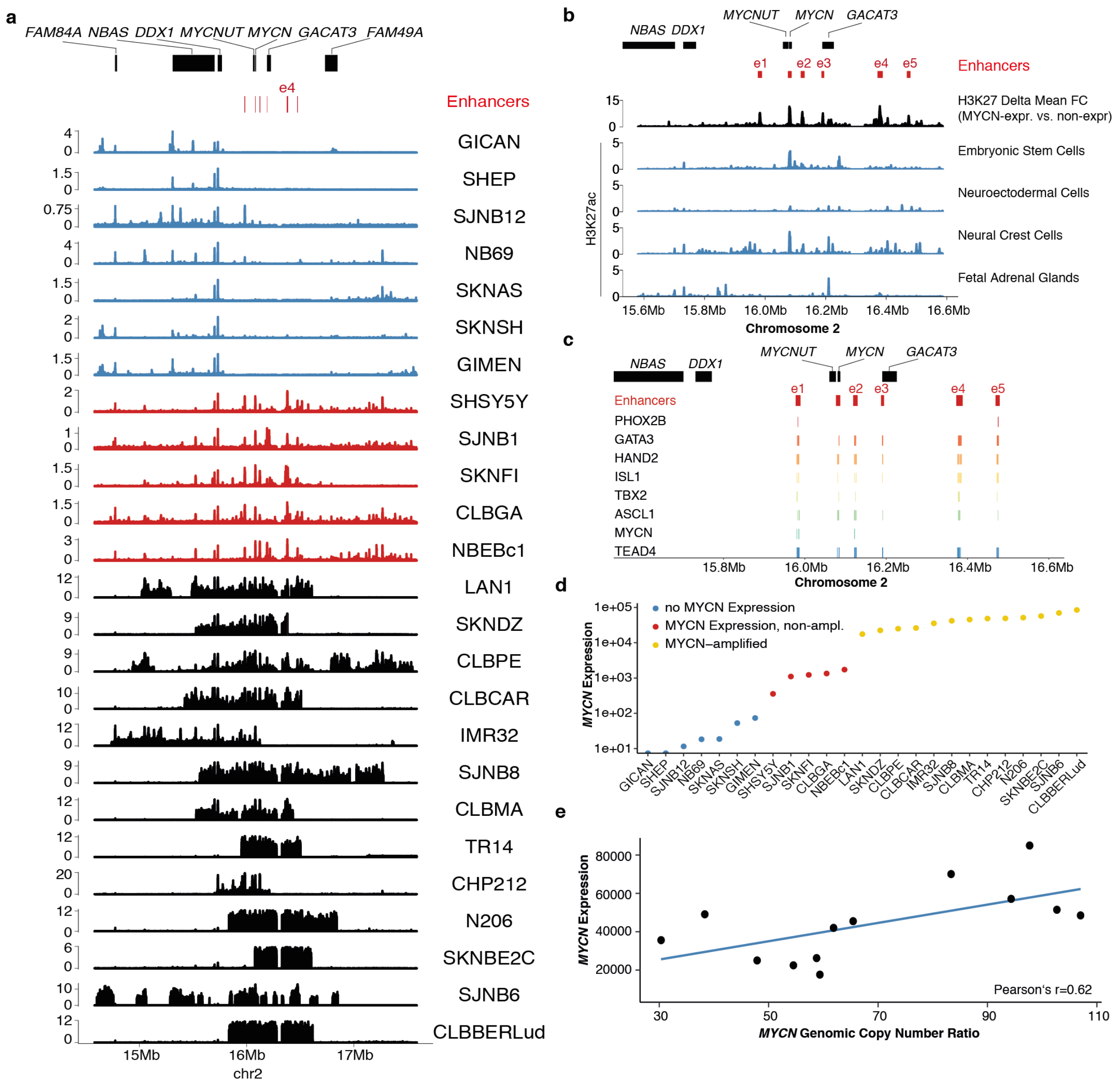

Supplementary Fig. 1. Enhancer profiling of the *MYCN* locus. a H3K27ac ChIP-seq signal (counts per million in 10bp bins, smoothed in 1kb bins) for seven non-*MYCN*-expressing neuroblastoma cell lines (blue), five *MYCN*-expressing non-*MYCN*-amplified neuroblastoma cell lines (red) and 13 MYCN-amplified neuroblastoma cell lines (black). **b** Differential composite H3K27ac signal for *MYCN*-non-expressing vs. *MYCN*-expressing non-*MYCN*-amplified cells (difference in the group-wise mean fold change H3K27ac vs. input; black) and H3K27ac ChIP-seq (counts per million in 10bp bins, smoothed in 1kb bins) signal for *in vitro* differentiated developmental cell types (embryonic stem cells, neuroectodermal cells, neural crest cells; blue) and a fetal adrenal cell sample (blue). **c** Core regulatory circuit factor (PHOX2B, GATA3, HAND2, ISL1, TBX2, ASCL1), MYCN and TEAD4 binding motif positions in the *MYCN*-driving enhancers e1-e5. Only motif hits within the enhancer regions are depicted. **d** *MYCN* expression for *N*=25 neuroblastoma cell lines as determined by RNA-seq (one sequencing experiment per cell line; size factor-normalized read counts) classified into no *MYCN* expression (size factor normalized expression lower than 100), *MYCN*-expressing non-*MYCN*-amplified cells (size factor normalized expression 100 or more) and *MYCN*-amplified cell lines. **e** *MYCN* expression by *MYCN* genomic copy number ratio determined from ChIP-seq input data for *N*=13 *MYCN*-amplified neuroblastoma cell lines (one sequencing experiment per cell line). Source data are provided as a Source Data file.

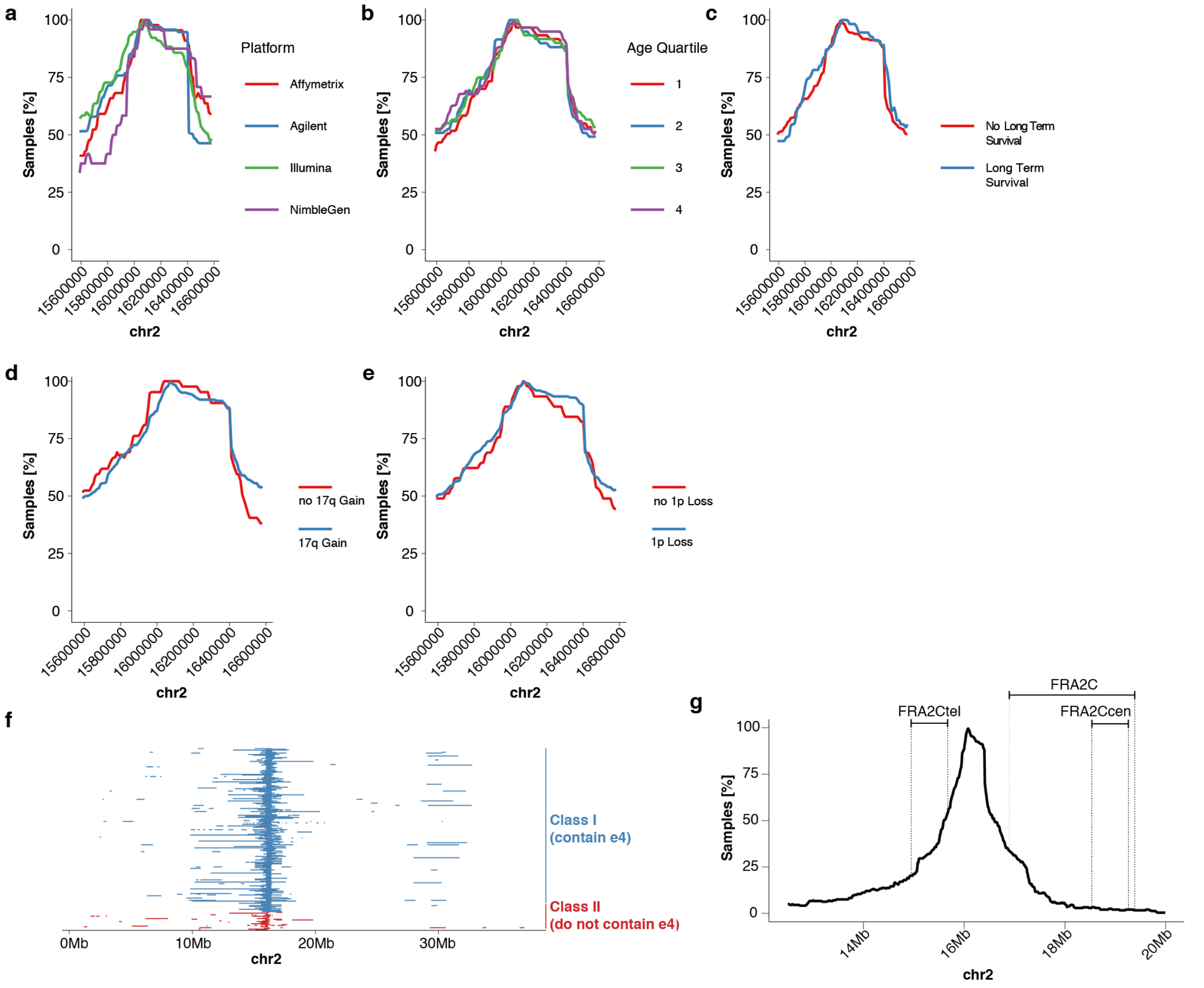

Supplementary Fig. 2. Amplification patterns across clinical and experimental covariates. **a-e** Percent co-amplification in 10kb bins for *MYCN*-amplified neuroblastoma (n=240) split by the experimental method to measure genomic copy-number (**a**, Affymetrix SNP array, Agilent aCGH platform, Illumina SNP array, NimbleGen aCGH platform), the age quartile of patients (**b**, 1=lowest quartile, 4=highest quartile), long-term survival (**c,** defined as survival beyond five years post diagnosis) and the genetic factors 17q gain **(d)** and 1p loss (**e**). **f** Amplified regions on chromosome 2 (0Mb-40Mb) for primary neuroblastoma (n=240), colored by amplicon class (class I amplicons including e4 vs. class II amplicons not including e4) **g** Percent co-amplification in 10kb bins around *MYCN* for *MYCN*-amplified neuroblastoma (n=240) and position of common fragile sites on chromosome 2 between 12Mb and 20Mb. Source data are provided as a Source Data file.

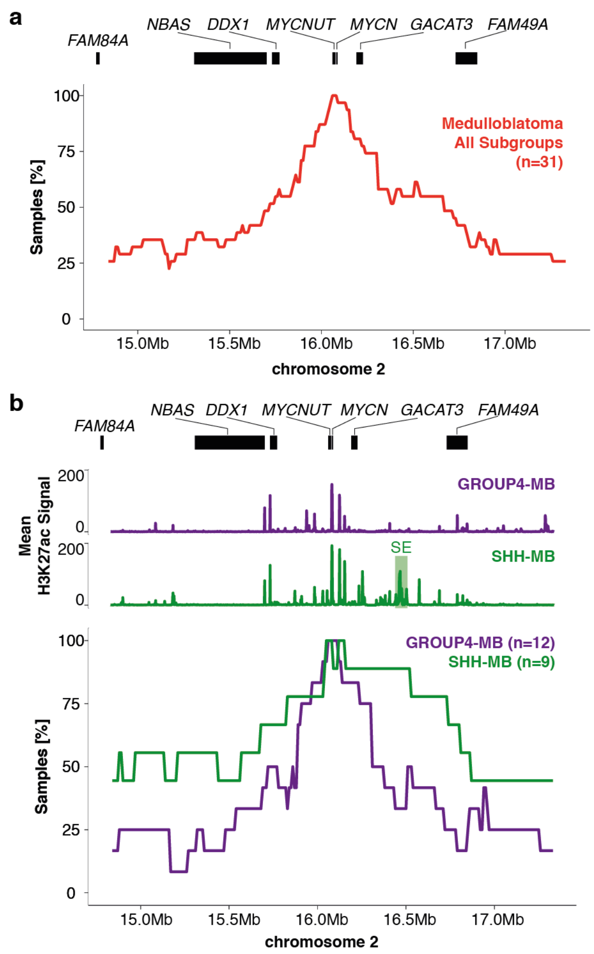

Supplementary Fig. 3. Super enhancer co-amplification confers different *MYCN* amplicon structures of medulloblastoma subgroups. a Percent co-amplification in 10kb bins for *MYCN*-amplified medulloblastoma across all medulloblastoma subgroups (n=31). **b** Mean H3K27ac ChIP-seq signal for GROUP4-MB (n=11) and SHH-MB (n=5) and percent co-amplification in 10kb bins for *MYCN*-amplified medulloblastoma for GROUP4-MB (n=12) and SHH-MB (n=9). The SHH-MB-specific super enhancer is marked by SE. Only medulloblastoma subgroups with more than five *MYCN* amplicons were considered for aggregate copy number profiles. Source data are provided as a Source Data file.

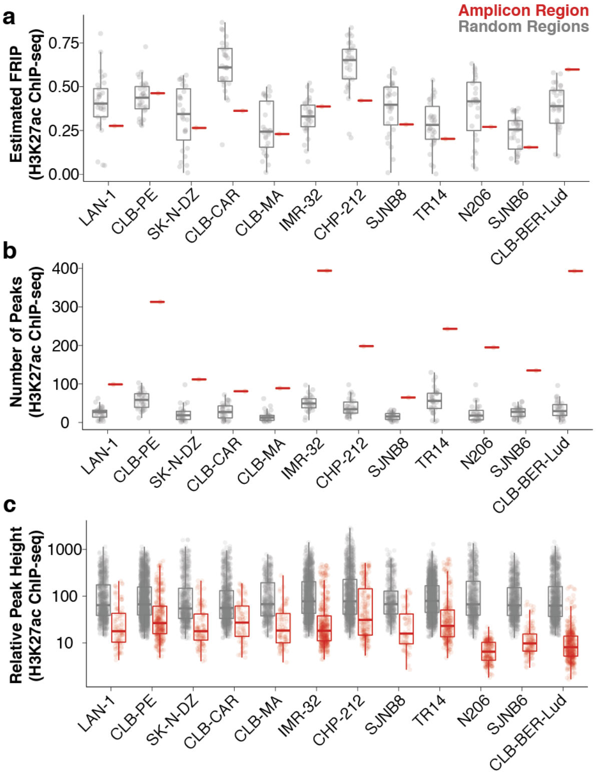

Supplementary Fig. 4. H3K27 acetylation on the *MYCN* amplicon. a Estimated fraction of H3K27ac reads in peaks for amplified regions vs. randomly drawn genomic regions of matching size (n=30) in 12 *MYCN*-amplified neuroblastoma cell lines. **b** Number of peaks for amplified regions (red) vs. randomly drawn genomic regions of matching size (grey, n=30) in 12 *MYCN*-amplified neuroblastoma cell lines. **c** Relative H3K27ac peak heights (compared to amplicon background) for amplified regions (red) vs. randomly drawn genomic regions of matching size (grey, n=30). In all boxplots, boxes depict the median, the upper quartile boundary and the lower quartile boundary. Whiskers extends to the largest data point within 1.5-fold of the inter-quartile range. Source data are provided as a Source Data file.

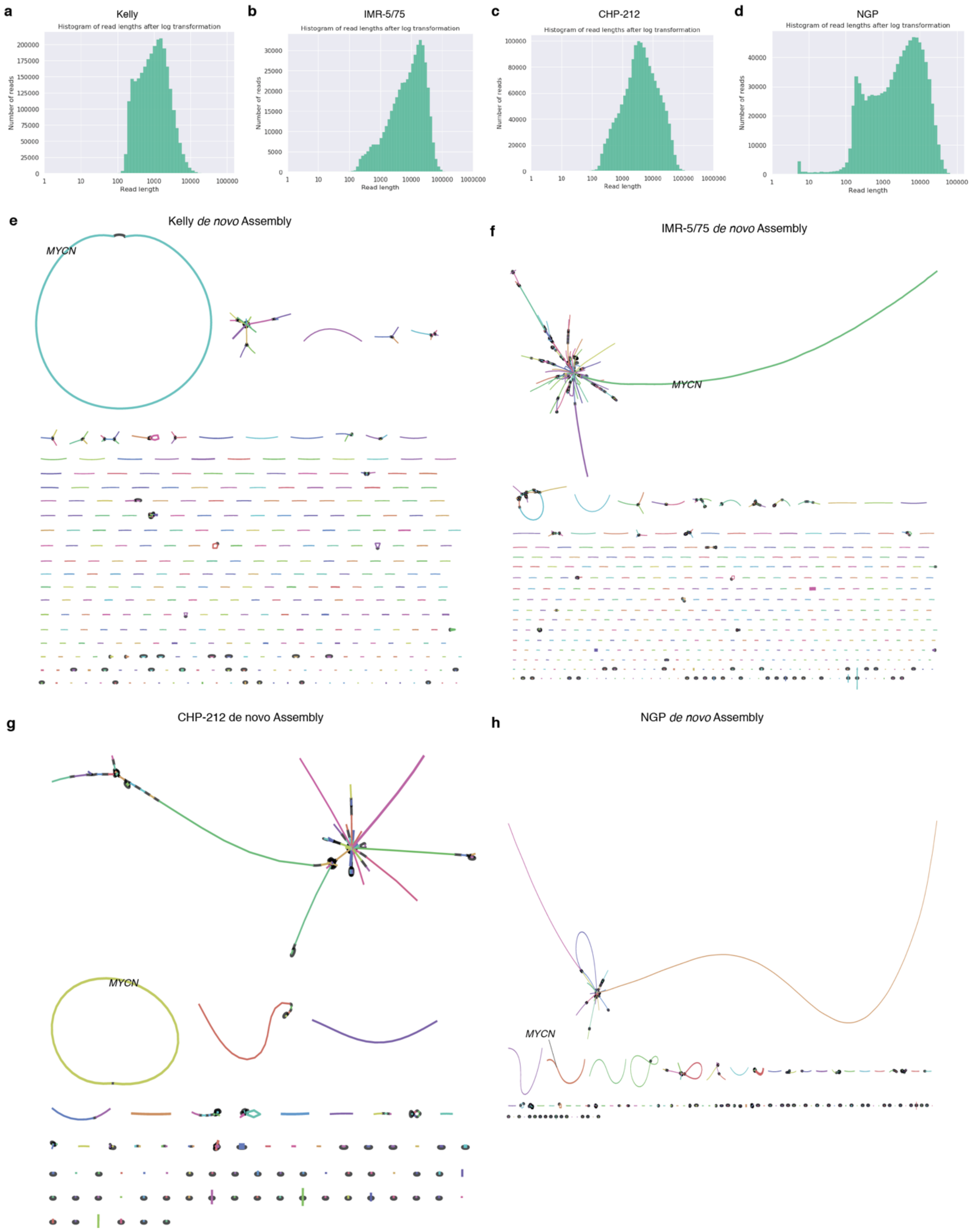

Supplementary Fig. 5. Long-read sequencing enables de novo assembly of *MYCN* neighborhoods. a-d Nanopore read length distribution (log-transformed) for the neuroblastoma cell lines Kelly (**a**, n=2,654,406), IMR-5/75 (**b**, n=474,980), CHP-212 (**c**, n=1,554,048) and NGP (**d**, n=952,031). **e** Nanopore long read-based de novo assembly of Kelly cells yields 477 contigs and an overall assembly N50 of 24,929 bp. BLAST analysis locates *MYCN* on a circular 975,932 bp contig. **f** Nanopore long read-based de novo assembly of IMR-5/75 cells yields 6,265 contigs and an overall assembly N50 of 91,273 bp. BLAST analysis locates *MYCN* on a linear 3,201,197 bp contig. **g** Nanopore long read-based de novo assembly of CHP-212 cells yields 21,264 contigs and an overall assembly N50 of 113,845 bp. BLAST analysis locates *MYCN* on a circular 1,705,218 bp contig. **h** Nanopore long read-based de novo assembly of NGP cells yields 6,550 contigs and an overall assembly N50 of 60,981 bp. BLAST analysis locates *MYCN* on a linear 623,907 bp contig.

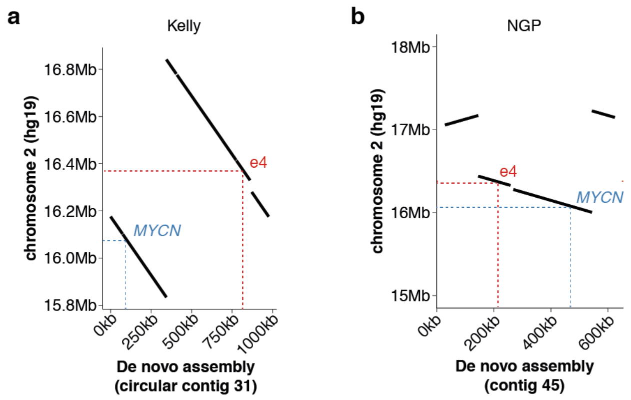

Supplementary Fig. 6. De novo assembly confirms co-amplification of *MYCN* and e4 in Kelly and NGP. Mapping of the de novo assembled *MYCN*-containing contig to hg19 in Kelly (**a**) and NGP (**b**) cells. Positions of *MYCN* and e4 are marked on the contig and in the reference genome. Note that the Kelly contig is circular such that the shortest distance from e4 to *MYCN* spans the contig circle junction.

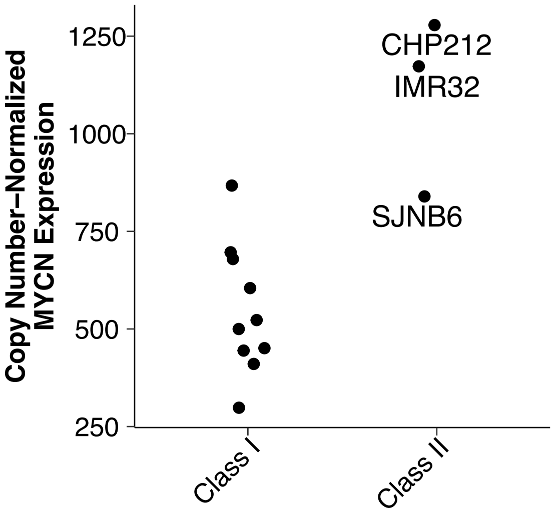

Supplementary Fig. 7. *MYCN* expression on two amplicon classes. *MYCN* copy number was estimated from ChIP-seq input signal for *N*=13 *MYCN*-amplified neuroblastoma cell lines, three of which were classified as class II (CHP-212, IMR-32, SJNB6). Size factor-normalized RNA-seq read counts from one sequencing experiment per cell line were divided by copy number ratios to estimate *MYCN* expression per genomic copy. This revealed relatively high expression per copy for class II amplicons although low sample size precludes further interpretation. Source Data are provided as a Source Data file.

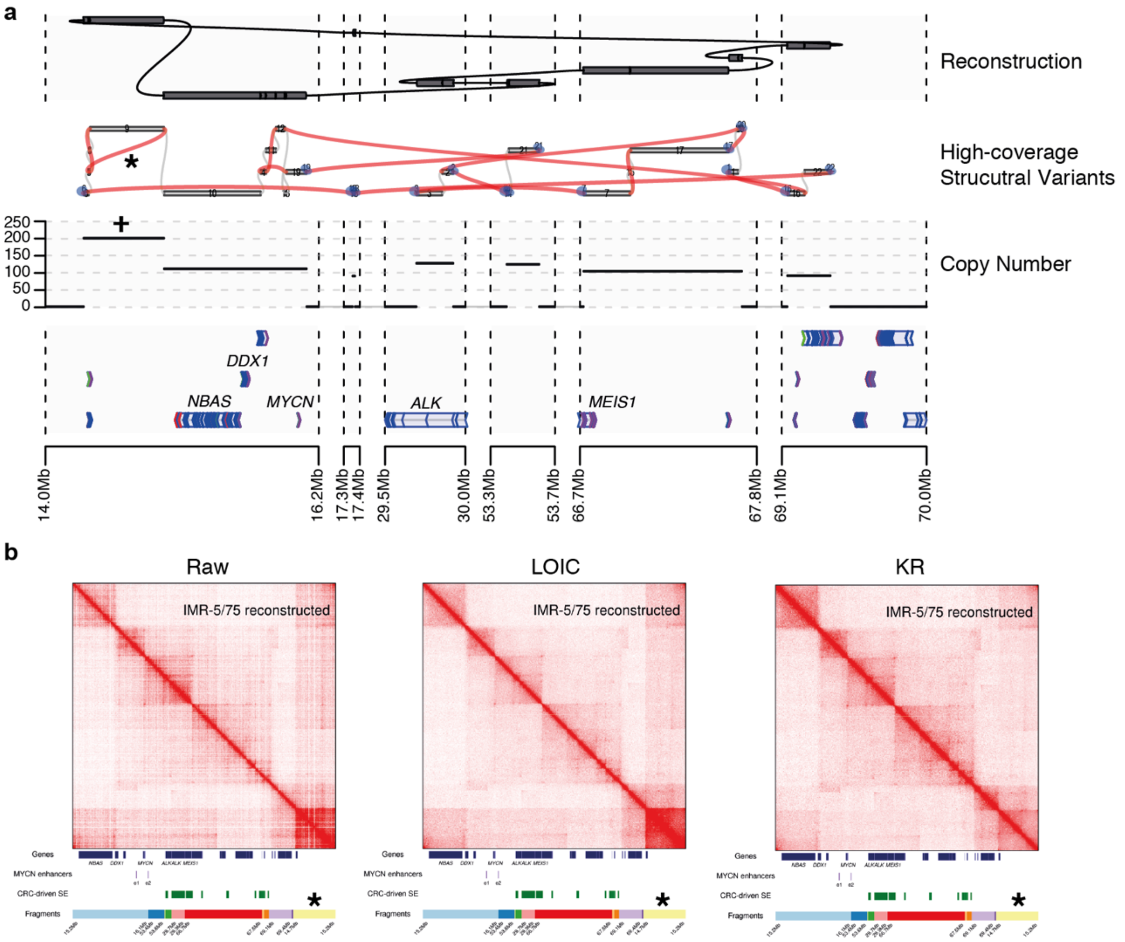

Supplementary Fig. 8. A tandem duplication on the *MYCN* amplicon in IMR-5/75. a Concordant copy-number (marked by a plus sign) and structural variation (marked by an asterisk) data indicates the presence of a tandem duplication on the *MYCN* amplicon in IMR-5/75. **b** Comparison of unnormalized, local iterative correction-normalized (LOIC; taking copy-number variation within the amplicon explicitly into account), as well as Knight-Ruiz-normalized (KR) Hi-C maps for the reconstructed amplicon shows that the finding of a neo-TAD is robust across different normalization approaches. The tandem duplication is marked by an asterisk.

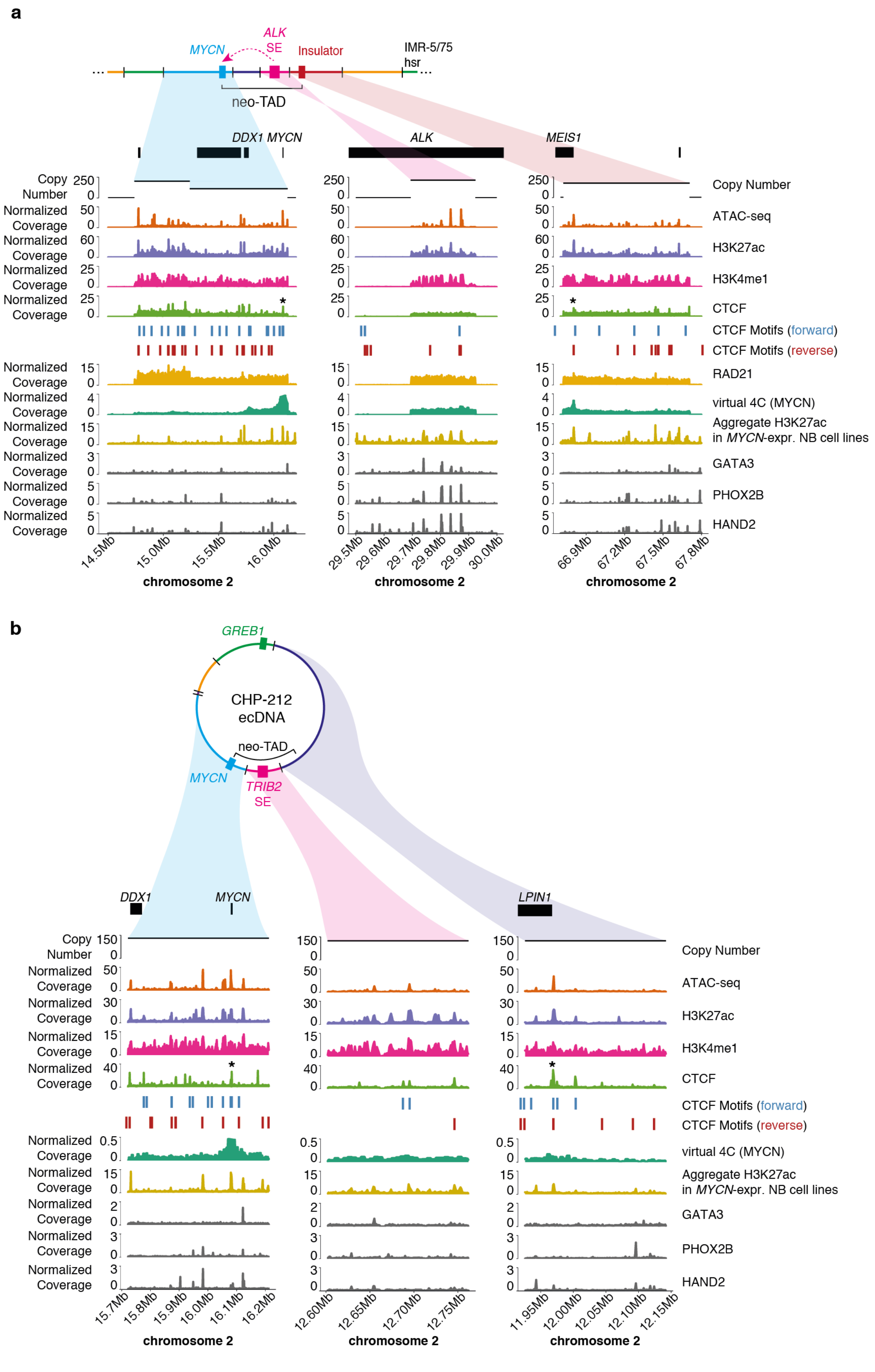

Supplementary Fig. 9. Enhancer hijacking and neo-TAD formation on the *MYCN* amplicon. Regions that contribute to neo-TAD formation are depicted for IMR-5/75 (**a**) and CHP-212 (**b**). Top to bottom: Gene bodies, Copy Number (black), ATAC-seq (orange), H3K27ac ChIP-seq (purple), H3K4me1 ChIP-seq (pink), CTCF ChIP-seq (light green), CTCF-

peak overlapping CTCF forward (blue) vs. reverse (red) binding motifs, RAD21 ChIP-seq (yellow; only available for IMR-5/75), virtual 4C with *MYCN* as the viewpoint (mean Knight-Ruiz normalized interaction frequency of three 5kb bins (chr2:16,075,000-16,085,000) around *MYCN*; dark green), the aggregate H3K27ac signal over 7 *MYCN*-expressing non-*MYCN*-amplified neuroblastoma cell lines (mean fold change over input; light brown), GATA3 ChIP-seq, PHOX2B ChIP-seq and HAND2 ChIP-seq (all CRC transcription factors in the neuroblastoma cell lines CLB-GA; grey). ChIP-seq and ATAC-seq is depicted as counts per million in 10bp bins, smoothed in 1kb bins. Neo-TAD boundaries are marked by an asterisk. Source data are provided as a Source Data file.

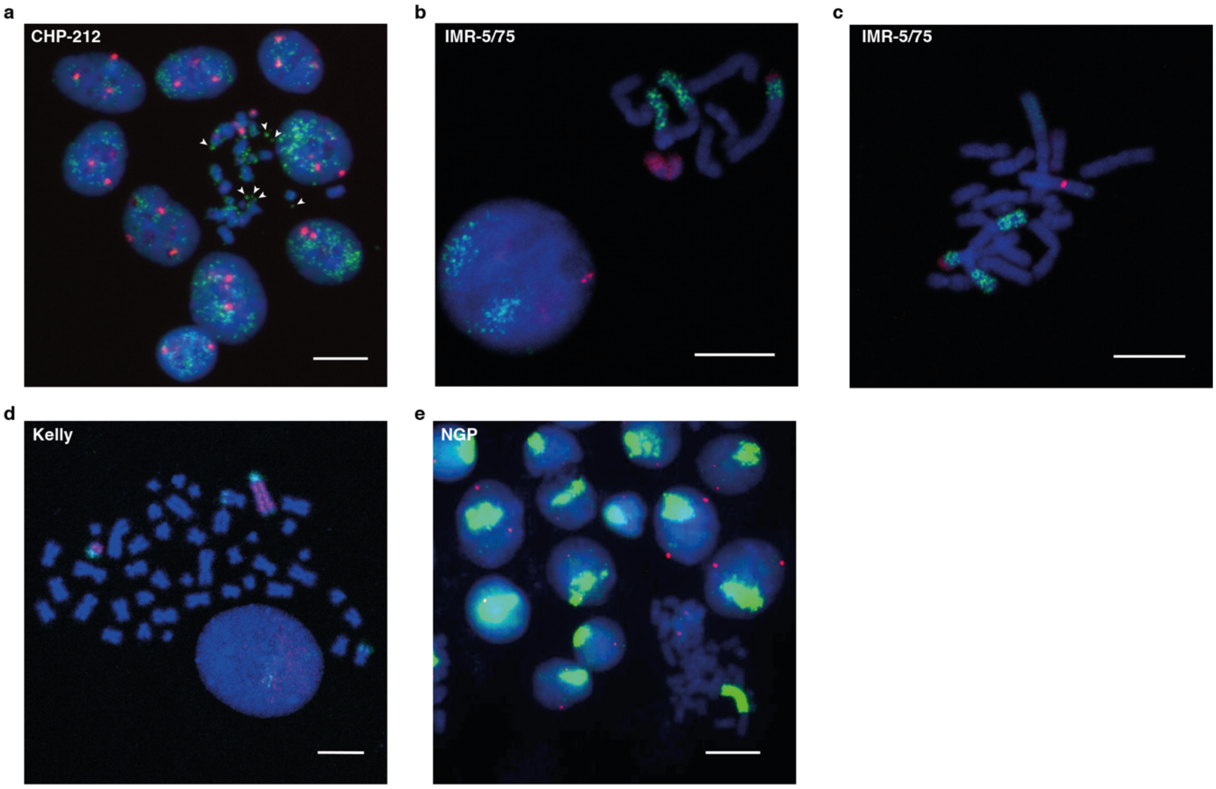

Supplementary Fig. 10. *MYCN* fluorescence in situ hybridization in neuroblastoma cell lines. **a** Fluorescence in situ hybridization (FISH) of CHP-212 metaphase spreads with a *MYCN* probe (green) and a probe for the chromosome 2 centromere (red). Arrowheads point to *MYCN* ecDNA. **b, c** FISH of metaphase spreads in IMR-5/75 with a *MYCN* probe (green), a chromosome 2 centromere (red) and a chromosome 12 paint (red) **d** FISH of metaphase spreads in Kelly cells with a *MYCN* probe (red) and chromosome 17 paint (green). **e** FISH of NGP metaphase spreads with a *MYCN* probe (green) and a probe for the chromosome 2 centromere (red). Scale bars are 10µm. FISH experiments were performed once per cell line.
