# Supplementary material for: Enhancer hijacking determines extrachromosomal circular MYCN amplicon architecture in neuroblastoma
Source: Nat Commun. 2020 Nov 16;11:5823. doi: 10.1038/s41467-020-19452-y (PMC7669906; doi:10.1038/s41467-020-19452-y)
Supplement: Supplementary file 2 — Reporting Summary [file 41467_2020_19452_MOESM2_ESM.pdf]

nature research

Corresponding author(s): Anton G. Henssen, Richard P. Koche

Last updated by author(s): Sep 19, 2020

# Reporting Summary

Nature Research wishes to improve the reproducibility of the work that we publish. This form provides structure for consistency and transparency in reporting. For further information on Nature Research policies, see Authors & Referees and the Editorial Policy Checklist.

## Statistics

For all statistical analyses, confirm that the following items are present in the figure legend, table legend, main text, or Methods section.

| n/a | Confirmed | |
|---|---|---|
| ☐ | ☒ | The exact sample size (*n*) for each experimental group/condition, given as a discrete number and unit of measurement |
| ☐ | ☒ | A statement on whether measurements were taken from distinct samples or whether the same sample was measured repeatedly |
| ☐ | ☒ | The statistical test(s) used AND whether they are one- or two-sided<br>*Only common tests should be described solely by name; describe more complex techniques in the Methods section.* |
| ☐ | ☒ | A description of all covariates tested |
| ☐ | ☒ | A description of any assumptions or corrections, such as tests of normality and adjustment for multiple comparisons |
| ☐ | ☒ | A full description of the statistical parameters including central tendency (e.g. means) or other basic estimates (e.g. regression coefficient) AND variation (e.g. standard deviation) or associated estimates of uncertainty (e.g. confidence intervals) |
| ☐ | ☒ | For null hypothesis testing, the test statistic (e.g. *F*, *t*, *r*) with confidence intervals, effect sizes, degrees of freedom and *P* value noted<br>*Give P values as exact values whenever suitable.* |
| ☒ | ☐ | For Bayesian analysis, information on the choice of priors and Markov chain Monte Carlo settings |
| ☒ | ☐ | For hierarchical and complex designs, identification of the appropriate level for tests and full reporting of outcomes |
| ☐ | ☒ | Estimates of effect sizes (e.g. Cohen's *d*, Pearson's *r*), indicating how they were calculated |

*Our web collection on statistics for biologists contains articles on many of the points above.*

## Software and code

Policy information about availability of computer code

| | |
|---|---|
| Data collection | No software was used for data acquisition. |
| Data analysis | FASTQC 0.11.8, BBMap 38.58, STAR 2.7.1, Subread package 1.6.4, DESeq2 1.22.2, BWA-MEM 0.7.15, Picard 2.20.4, Deeptools 3.3.0, MACS2 2.1.2, Phantompeakqualtools 1.2.1, Juicer pipeline v1.5.6 CPU version, BWA v0.7.17, Juicer tools v1.7.5, Control-FREEC 11.4, SvABA 1.1.1, Guppy 2.3.7, Flye 2.4.2, NanoPlot 1.0.0, Bandage 0.8.1, Ribbon 1.0, Megalodon 0.1.0, R 3.5.1, Rawcopy 1.1, QDNAseq 1.22.0, TFBSTools 1.20.0, minimap2 2.16<br><br>Code is available at https://github.com/henssenlab/MYCNAmplicon. |

For manuscripts utilizing custom algorithms or software that are central to the research but not yet described in published literature, software must be made available to editors/reviewers. We strongly encourage code deposition in a community repository (e.g. GitHub). See the Nature Research guidelines for submitting code & software for further information.

## Data

Policy information about availability of data

All manuscripts must include a data availability statement. This statement should provide the following information, where applicable:

- Accession codes, unique identifiers, or web links for publicly available datasets
- A list of figures that have associated raw data
- A description of any restrictions on data availability

Sequencing data generated for this study are available at the Sequence Read Archive under accession PRJNA622577. Copy number data for high-risk neuroblastoma were downloaded from https://github.com/padpuydt/copynumber_HR_NB/27. Public data supporting the findings of this manuscript were downloaded from the Gene Expression Omnibus under accessions GSE90683, GSE80152, GSE24447, GSE37385, GSE18927 and GSE28874 and from ArrayExpress under accession E-MTAB-6570. Medulloblastoma ChIP-seq data were downloaded from https://pecan.stjude.cloud/dataset/northcott. Corresponding BigWig und narrowPeak files can be downloaded from https://data.cyverse.org/dav-anon/iplant/home/konstantin/helmsaueretal/. An accompanying UCSC genome browser track hub is provided for

ChIP-seq and ATAC-seq data visualization (https://de.cyverse.org/dl/d/27AA17DA-F24C-4BF4-904C-62B539A47DCC/hub.txt). Source data are provided with this paper. The source data underlying Fig. 1-4 and 6, Supplementary Figs. 1, 3, 4 and 7 are provided as a Source Data file. All other data is available from the corresponding authors upon reasonable request.

# Field-specific reporting

Please select the one below that is the best fit for your research. If you are not sure, read the appropriate sections before making your selection.

☒ Life sciences ☐ Behavioural & social sciences ☐ Ecological, evolutionary & environmental sciences

For a reference copy of the document with all sections, see nature.com/documents/nr-reporting-summary-flat.pdf

# Life sciences study design

All studies must disclose on these points even when the disclosure is negative.

| | |
|---|---|
| Sample size | The sample size for copy number data was given by the sample size of the public data. We chose four cell lines as in-depth case studies. We did not test specific hypotheses with these data for which sample size calculation would have been applicable but rather describe two cases of enhancer hijacking in class II amplicons. |
| Data exclusions | No data was excluded. |
| Replication | For large parts of our analysis, we used published sequencing data that had not been acquired in replicates. When we acquired ChIP-seq/ ATAC-seq data ourselves, this was not done with replicates. Hi-C data was acquired in technical duplicates and merged after individual inspection of the data. FISH experiments were done once per cell line. |
| Randomization | Not applicable. The study did not have different experimental groups. |
| Blinding | Not applicable. The study did not have experimental groups such that investigators could not be blinded to group allocation. |

# Reporting for specific materials, systems and methods

We require information from authors about some types of materials, experimental systems and methods used in many studies. Here, indicate whether each material, system or method listed is relevant to your study. If you are not sure if a list item applies to your research, read the appropriate section before selecting a response.

## Materials & experimental systems

| n/a | Involved in the study | |
|---|---|---|
| ☐ | ☒ | Antibodies |
| ☐ | ☒ | Eukaryotic cell lines |
| ☒ | ☐ | Palaeontology |
| ☒ | ☐ | Animals and other organisms |
| ☒ | ☐ | Human research participants |
| ☒ | ☐ | Clinical data |

## Methods

| n/a | Involved in the study | |
|---|---|---|
| ☐ | ☒ | ChIP-seq |
| ☒ | ☐ | Flow cytometry |
| ☒ | ☐ | MRI-based neuroimaging |

## Antibodies

| | |
|---|---|
| Antibodies used | Anti-H3K27ac (Diagenode c15410174; lot A7071-001P; dilution 1:500)<br>anti-H3K4m1 (Abcam; ab8895; lot GR141677-1; dilution 1:1200)<br>anti-RAD21 (Abcam; ab992; lot GR221348-8; dilution 1:150)<br>anti-CTCF (Active Motif; 613111; lot 34614003; dilution 1:150) |
| Validation | Anti-H3K27ac (Diagenode c15410174; lot A7071-001P) has been validated for ChIP by the manufacturer (see https://www.diagenode.com/en/p/h3k27ac-polyclonal-antibody-classic-50-mg-42-ml)<br>Anti-H3K4m1 (Abcam; ab8895; lot GR141677-1) has been validated for ChIP by the manufacturer (see https://www.abcam.com/histone-h3-mono-methyl-k4-antibody-chip-grade-ab8895.html)<br>Anti-CTCF (Active Motif; 613111; lot 34614003) has been validated for ChIP by the manufacturer (see https://www.activemotif.com/catalog/details/61311/ctcf-antibody-pab)<br>Anti-RAD21 (Abcam; ab992; Lot GR221348-8) has been used in several publications for Rad21 ChIP. See references at https://www.abcam.com/rad21-antibody-ab992.html, e.g. Khoury et al. Nature Communications 2020, Yameda et al. Nature 2019 or publications from our own lab, e.g. Jerković et al 2017. |

# Eukaryotic cell lines

Policy information about cell lines

| | |
|---|---|
| Cell line source(s) | Neuroblastoma cell lines were a gift from F. Speleman (Cancer Research Institute Ghent, Ghent, Belgium; NGP), F. Westermann (German Cancer Research Center, Heidelberg, Germany; IMR-5/75), obtained from the German Collection of Microorganisms and Cell Cultures (DSMZ GmbH, Braunschweig, Germany; Kelly) or obtained from the American Type Culture Collection (ATCC, Manassas, VA; CHP-212). |
| Authentication | Cell line identity was verified by STR genotyping (Genetica DNA Laboratories, Burlington, NC and IDEXX BioResearch, Westbrook, ME). |
| Mycoplasma contamination | Absence of Mycoplasma sp. contamination was determined with a Lonza MycoAlert system (Lonza Group Ltd., Basel, CH). |
| Commonly misidentified lines (See ICLAC register) | No commonly misidentified lines were used in this study. |

# ChIP-seq

## Data deposition

☒ Confirm that both raw and final processed data have been deposited in a public database such as GEO.

☒ Confirm that you have deposited or provided access to graph files (e.g. BED files) for the called peaks.

| | |
|---|---|
| Data access links *May remain private before publication.* | Sequencing data generated for this study are available at the Sequence Read Archive under accession PRJNA622577. Copy number data for high-risk neuroblastoma were downloaded from https://github.com/padpuydt/copynumber_HR_NB/27. Public data supporting the findings of this manuscript were downloaded from the Gene Expression Omnibus under accessions GSE90683, GSE80152, GSE24447, GSE37385, GSE18927 and GSE28874 and from ArrayExpress under accession E-MTAB-6570. Medulloblastoma ChIP-seq data were downloaded from https://pecan.stjude.cloud/dataset/northcott. Corresponding BigWig und narrowPeak files can be downloaded from https://data.cyverse.org/dav-anon/iplant/home/konstantin/helmsaueretal/. An accompanying UCSC genome browser track hub is provided for ChIP-seq and ATAC-seq data visualization (https://de.cyverse.org/dl/d/27AA17DA-F24C-4BF4-904C-62B539A47DCC/hub.txt). Source data are provided with this paper. The source data underlying Fig. 1-4 and 6, Supplementary Figs. 1, 3, 4 and 7 are provided as a Source Data file. All other data is available from the corresponding authors upon reasonable request. |
| Files in database submission | See https://data.cyverse.org/dav-anon/iplant/home/konstantin/helmsaueretal/ |
| Genome browser session (e.g. UCSC) | A track hub for the UCSC genome browser is available (https://de.cyverse.org/dl/d/27AA17DA-F24C-4BF4-904C-62B539A47DCC/hub.txt) |

## Methodology

| | |
|---|---|
| Replicates | This study is mainly based on published ChIP-seq data which had not been acquired in replicates. For additional data acquired, this was not performed with replicates. Peak statistics were initially derived from comparison to the read distribution in matched input samples, and then compared between samples, as described in detail in the Methods. Hi-C was acquired in technical duplicates and merged after inspection. |
| Sequencing depth | ChIP-seq: 25M 75bp single-end reads |
| Antibodies | Anti-H3K27ac (Diagenode c15410174; lot A7071-001P; dilution 1:500)<br>anti-H3K4m1 (Abcam; ab8895; lot GR141677-1; dilution 1:1200)<br>anti-RAD21 (Abcam; ab992; lot GR221348-8; dilution 1:150)<br>anti-CTCF (Active Motif; 613111; lot 34614003; dilution 1:150) |
| Peak calling parameters | MACS2 (2.1.2) with default parameters |
| Data quality | Data was quality controlled using RPC, NPC and composite plots over housekeeping genes. |
| Software | FASTQC 0.11.8, BBMap 38.58, BWA-MEM 0.7.15, Picard 2.20.4, Deeptools 3.3.0, MACS2 2.1.2 |
